# Structural and biochemical characterization of the biuret hydrolase (BiuH) from the cyanuric acid catabolism pathway of *Rhizobium leguminasorum* bv. *viciae* 3841

**DOI:** 10.1371/journal.pone.0192736

**Published:** 2018-02-09

**Authors:** Lygie Esquirol, Thomas S. Peat, Matthew Wilding, Del Lucent, Nigel G. French, Carol J. Hartley, Janet Newman, Colin Scott

**Affiliations:** 1 CSIRO Biocatalysis and Synthetic Biology, Canberra, Australian Capital Territory, Australia; 2 Research School of Chemistry, Australian National University, Canberra, Australian Capital Territory, Australia; 3 CSIRO Biomedical Manufacturing, Parkville, Melbourne, Victoria, Australia; 4 Department of Electrical Engineering and Physics, Wilkes University, Wilkes-Barre, Pennsylvania, United States of America; University of Canterbury, NEW ZEALAND

## Abstract

Biuret deamination is an essential step in cyanuric acid mineralization. In the well-studied atrazine degrading bacterium *Pseudomonas* sp. strain ADP, the amidase AtzE catalyzes this step. However, *Rhizobium leguminosarum* bv. *viciae* 3841 uses an unrelated cysteine hydrolase, BiuH, instead. Herein, structures of BiuH, BiuH with bound inhibitor and variants of BiuH are reported. The substrate is bound in the active site by a hydrogen bonding network that imparts high substrate specificity. The structure of the inactive Cys175Ser BiuH variant with substrate bound in the active site revealed that an active site cysteine (Cys175), aspartic acid (Asp36) and lysine (Lys142) form a catalytic triad, which is consistent with biochemical studies of BiuH variants. Finally, molecular dynamics simulations highlighted the presence of three channels from the active site to the enzyme surface: a persistent tunnel gated by residues Val218 and Gln215 forming a potential substrate channel and two smaller channels formed by Val28 and a mobile loop (including residues Phe41, Tyr47 and Met51) that may serve as channels for co-product (ammonia) or co-substrate (water).

## Introduction

The mineralization of cyanuric acid by bacteria is thought to be an ancient metabolic pathway [1]. It is thought that this pathway has been recently ‘co-opted’ into pathways for the degradation of highly functionalized *s*-triazines as they have become environmentally abundant through human activities since the mid-twentieth century [1–3]. The s-*triazine* mineralization pathways, including the cyanuric acid catabolism pathway, are thought to have evolved in response to an increase in the abundance of *s-*triazines in the environment as a result of human activities [2,4,5]. Although most incidentally exposed bacteria are not sensitive to the *s-*triazines, these anthropogenic compounds are an excellent nitrogen source and bacteria that can access this nitrogen may have a growth advantage compared with those that cannot: i.e., the ability to use *s-*triazines as a nitrogen source confers a selective advantage [6].

**Fig 1:**
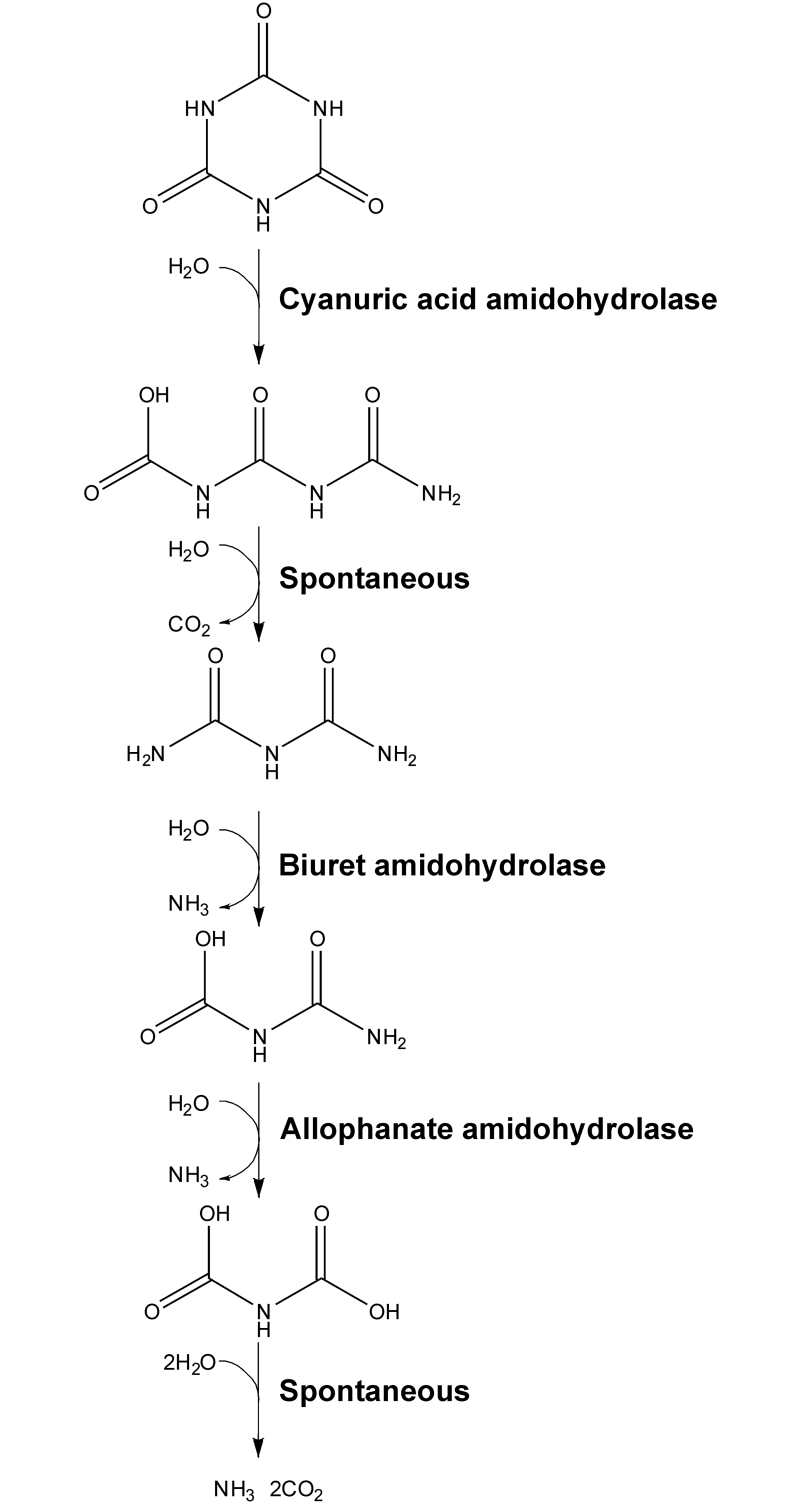
Cyanuric acid mineralization by bacteria. Cyanuric acid is mineralized to CO_2_ and NH_3_ by cyanuric acid mineralizing bacteria by the enzymes cyanuric acid amidohydrolase, biuret hydrolase and allophanate hydrolase. The product of cyanuric acid amiidohydrolase (1-carboxybiuret) is unstable under physiological conditions and decarboxylates to form biuret and CO_2_. The product of allophanate amidohydrolase (dicarboxyammonia) is also unstable under physiological conditions and decomposes to CO_2_ and NH_3_.

The canonical cyanuric acid catabolism pathway was first described from the atrazine-degrading *Pseudomonas* sp. strain ADP, which converts herbicidal chloro-*s*-triazines to cyanuric acid *via* three sequential hydrolyses that first dechlorinate and then dealkylate the herbicides. These steps are catalyzed by AtzA [7], AtzB [8] and AtzC [9], all of which are metalloenzymes with an amidohydrolase fold (PFAM PF01979). Cyanuric acid is then mineralized by three hydrolases (Fig 1): AtzD [10–12], AtzE [13,14] and AtzF [15–18]. AtzE and AtzF are Ser-*cis*Ser-Lys amidohydrolases and both possess an amidase-fold (PFAM PF01425); the structure of AtzF has been determined experimentally [15,19], while that of AtzE has been inferred from its sequence identity with other amidase proteins [14]. AtzD is also a serine hydrolase, but it belongs to a recently described structural family (the Toblerone fold; PFAM PF09663) that is unrelated to Ser-*cis*Ser-Lys hydrolases [10,11,20].

Other *s-*triazine catabolizing enzymes and pathways have also evolved in response to the increased abundance of *s-*triazines in the environment. For example, AtzA is substituted for a physiologically isofunctional, but non-homologous amidohydrolase TrzN in some bacterial species [21–23]. TriA, a melamine aminohydrolase that is 98% identical to, but biochemically distinct from AtzA, allows the use of the triamino triazine melamine as a nitrogen source in *Pseudomonas* sp. strain NRRL B-12227 [24–26]. *Rhizobium leguminosarum* bv. *viciae* 3841 has an unusual cyanuric acid catabolic pathway that uses an *atzD* homolog to ring-open cyanuric acid, but lacks an *atzE* homolog to catalyze the subsequent step [27]. Instead, *R*. *leguminosarum* bv. *viciae* 3841 employs a cysteine hydrolase that fulfils the role of a biuret amidohydrolase (BiuH). The genes encoding the cyanuric acid amidohydrolase and biuret hydrolase are co-located on one of six large plasmids (the ~0.5 Mbp ‘symbiosis’ plasmid, pRL10 [27]), albeit this location may not have a specific functional consequence as most *s-*triazine-degradation genes are associated with mobile genetic elements [2,5].

Potential applications for many of the genes and enzymes of the *s*-triazine degrading pathway have been identified, including environmental bioremediation and the detection of adulterants in food and animal feed [28–32]. Biuret is sometimes used as an inexpensive adulterant to increase the total nitrogen content of food and feed, thereby increasing its value [33]. Biuret is also a side product in the production of urea fertilizer and can be phytotoxic [34,35]. BiuH may have utility in detecting, quantifying and degrading biuret in food, feed and fertilizer.

Herein, we describe and characterize the structure and catalytic mechanism of BiuH. Through a combination of structural studies, mutagenesis and molecular modelling we are able to propose a plausible catalytic mechanism for BiuH, which closely resembles that of other cysteine hydrolases. We also identify three potential channels from the active site to the bulk solvent, for the ingress of substrates (biuret and water) to the active site and the egress of products (ammonia and allophanate).

## Materials and methods

### Cloning

A synthetic version of the biuret hydrolase gene (*biuH*) from *Rhizobium leguminasorum* bv. *viciae* 3841, codon optimized for expression in *E*. *coli*, encoding for a protein identical to Q1M7F4 (Uniprot), EMBL database accession no. AM236084.1 was ordered from GenScript (Piscataway, NJ, USA; S1 Fig) and provided as an insert in pUC57 (Genscript) with *Nde*I and *Bam*HI (New England Biolabs) restriction sites engineered 3’ and 5’, respectively, of the structural gene. The gene was subcloned into the *Nde*I and *Bam*HI sites of pETcc2, described in Peat *et al*., 2013. A sequence coding for a 6xHis-tag before a thrombin cleavage site (MGSSHHHHHHSSGLVPRGSH; S1 Fig) was introduced by the subcloning to facilitate protein purification.

Genes encoding single amino acid substitution variants of BiuH (Asp36Ala, Asp36Asn, Asp36Gln, Asp36Glu, Phe41Ala, Phe41Leu, Phe41Tyr, Phe41Trp, Lys142Ala, Lys142His and Lys142Arg, Lys145Ala Lys145His, Lys145Arg, Cys175Ala, Cys175Ser, Gln215Ala, Gln215Asn, Gln215Asp and Gln215GluGln215Glu) were produced by overlap extension PCR as described in Ho *et al* [36]. The *biuH* gene was used as template and mutagenic primers were obtained from Integrated DNA Technologies (IDT, Singapore) and their sequences are detailed in S1 Table. Mutant *biuH* genes were cloned into the pETcc2 expression vector using *Nde*I and *Bam*HI.

### Protein expression and purification

pETcc2 derivatives encoding the wild-type biuret hydrolase and twenty variants were used to transform *Escherichia coli* BL21 (λDE3) cells (New England Biolabs). Bacterial cultures were grown on Luria-Bertani (LB) medium, supplemented with 100 μg/mL ampicillin where required. Cells were grown with shaking at 200 rpm at 37 °C for the wild-type biuret hydrolase and 28 °C for the variants. Protein expression was induced at an OD_600_ of 0.8 by addition of 1 mM isopropyl β–D-1-thiogalactopyranoside (IPTG).

A seleno-L-methionine (SeMet)-BiuH was obtained by growth in minimal medium containing 60 mg/L of SeMet as the only source of methionine, as described in Doublié 1997 [37]. Purification was performed as described below, except that 0.2 mM EDTA and 5 mM dithiothreitol (DTT) were added to all the buffers in order to retard oxidation of the SeMet-BiuH during purification.

Cells were harvested 24 hours after induction by centrifugation at 5,000 x *g* for 15 minutes, resuspended in lysis buffer (5 mM imidazole, 25 mM potassium phosphate pH 7.5) and lysed by passage through a Microfluidics homogenizer M-110P (Massachusetts, USA) five times at 15,000 PSI. The lysis was followed by centrifugation at 18,000 x *g* for 45 minutes, using an Aventi J-E centrifuge to pellet the cells debris and the soluble fraction was used for further purification.

The soluble fraction was syringe filtered through a 0.22 μm filter. The filtrate was applied to a 5 mL Ni-NTA Superflow cartridge (GE Healthcare) and protein eluted with a gradient from 5 mM to 500 mM imidazole in ten column volumes (CV). SDS-PAGE analysis was performed to assess the purity of the fractions, using NuPAGE^®^ Novex 4–12% acrylamide gradient Bis-Tris gels (Invitrogen). Fractions that eluted between 150–350 mM imidazole were found to contain a protein with a mass corresponding to that of biuret hydrolase.

After pooling, the protein containing fractions were concentrated to 12 mL using an Amicon Ultra-15 centrifugal filter unit and further purified by size exclusion chromatography using a 130 mL column packed with Superdex 200 preparation grade resin (GE Healthcare Life Sciences), equilibrated initially with 50 mM HEPES pH 7.5, 100 mM NaCl. After a preliminary results from the differential scanning fluorimetry (DSF) the gel filtration buffer was swapped to one containing 50 mM Tris pH 7.5, 100 mM NaCl. All chromatography steps were performed using an ÄKTA purifier UPC 10 (GE Healthcare Life Sciences).

Protein concentration was estimated using NanoDrop spectrophotometer (Thermo Pierce) by reading the absorbance at λ = 280 nm. The molar extinction coefficients used for BiuH and its variants was 34,295 M^-1^.cm^-1^, except for BiuH Phe41Tyr (25,785 M^-1^.cm^-1^) and BiuH Phe41Trp (39,795 M^-1^.cm^-1^). Molar extinction coefficients were calculated using ProtParam on the ExPasy server (https://web.expasy.org/protparam/).

### Differential scanning fluorimetry (DSF)

DSF was used to determine an appropriate formulation for crystallization trials, using a standard, published protocol [38]. Briefly, 0.3 uL of protein and 0.3 uL of a 1:20 dilution of Sypro dye (Sigma S5692) was diluted into a final volume of 20 uL, and heated in steps of 0.5 °C from 20–90 °C in an RT-PCR machine (BioRad CXF 96). The protein at 3.6 mg/mL in a preliminary size exclusion formulation (50 mM HEPES pH 7.5, 100 mM NaCl) was tested in triplicate against an array of 13 different buffers at pH values ranging from 5 to 9, at two concentrations of NaCl (‘Buffer Screen 9’,). The T_m_ in the HEPES/NaCl formulation was 47.8+/-0.1 °C (all T_m_ estimations were extracted using the program Meltdown [39]). The protein was slightly more stable in 50 mM Tris chloride pH 8, 50 mM NaCl (51.5+/- 0.2 °C). The protein was simultaneously treated with thrombin to remove the N-terminal His-tag (100 uL protein at 3.6 mg/mL was added to a 0.2 mL tube containing 10 units of lyophilised thrombin, and enough CaCl_2_ to give a final concentration of 3 mM). This mix was dialyzed into 50 mM Tris pH 8, 50 mM NaCl (3.5 kDa cutoff membrane) and this sample was assayed without further purification against the same buffer screen by DSF. This increased the T_m_ to 55.1 °C. A Meltdown report for the Buffer Screen 9 analysis of the thrombin/tris treated protein is included as supplementary information (S2 Fig).

To confirm the proper folding of the BiuH variants, thermal melt analyses were performed on each variant, using the N-terminal His tagged protein. Protein concentration was 5 mg/mL, in 50 mM Tris chloride pH 7.5, 100 mM NaCl and 0.2 μL of 1:20 diluted Sypro Orange dye was added to each 20 μL experiment. The samples were run in 3–8 fold replication. The wild-type protein shows a T_m_ of around 56 °C, similar to that found from buffer screen 9, the variants ranged from 41 °C to 68 °C, but all showed a clear melting transition. See S2 Table and S3 Fig.

### Protein crystallization and structure solution

Modestly diffracting (≈3Å) wild type BiuH crystals suitable for X-ray analysis were eventually grown using a combination of seeding, *in situ* proteolysis, formulation variation and additive screening. The best native crystal tested was a thin, stacked plate grown from protein at 3 mg/mL in Tris/NaCl buffer, with an *in situ* chymotrypsin treatment, where 100 uL protein solution was added to 10 μg of freeze-dried chymotrypsin and this mix was set up with no further purification. The reservoir consisteded of 0.18 M lithium chloride, 0.3 M NDSB 195 (non-detergent sulfobetaine 195), 18% polyethylene glycol 6000, 0.09 M sodium MES pH 6. The crystals grew in droplets of 200 nL protein + 200 nL reservoir, and were set up in SwissSci SD2 sitting drop plates (Molecular Dimensions, UK) at 20 °C. As there was no obvious molecular replacement model available, SeMet protein in 50 mM Tris pH 8, 50 mM NaCl, 5 mM DTT at 10 mg/mL was set up against an optimization screen based around the successful wild-type BiuH condition, with the same *in situ* chymotrypsin treatment. A SeMet crystal from this optimization screen was harvested, and used to collect single wavelength anomalous data at 0.97919 wavelength to 2.46 Å resolution at the Australian Synchrotron MX2 beamline. The data showed a strong anomalous signal (CCanom > 0.15) to about 2.90 Å and the structure was solved using Crank2 [40] which automatically built 884 residues in 19 chains in the C2 spacegroup. The structure was manually rebuilt to give four independent chains in the asymmetric unit which formed a tight tetramer. Several higher resolution data sets (Table 1) of mutants and soaks were available and this model was used to solve these structures using Phaser [41] (in two new space groups, P2_1_2_1_2_1_ and P22_1_2_1_). The P22_1_2_1_ spacegroup (the two K142 mutant structures) also has a single tetramer in the asymmetric unit whereas the P2_1_2_1_2_1_ spacegroup (the C175S structure with and without biuret) has two tetramers in the asymmetric unit. The structures were manually rebuilt using Coot [42] and refined with Refmac [43]. The biuret and inhibitor constraints were generated with the eLBOW function in Phenix [44]. All the crystal trials were set up in the SD-2 sitting drop plates which were used for the native protein; details of the crystallization conditions can be found in S3 Table. The structures obtained in this study have been lodged in the Protein Data Bank: SeMet BiuH wild-type (PDB: 6AZO), BiuH Cys175Ser (PDB: 6AZN), BiuH Cys175Ser with biuret (PDB: 6AZQ), BiuH Lys142Ala with *N*-carbamoyl-D,L-aspartic acid (PDB: 6AZS) and BiuH Lys142His (PDB: 5BK6).

**Table 1:** Data collection and refinement statistics.

Data Collection					
PDB code	6AZO	6AZN	6AZQ	6AZS	5BK6
Crystal	SeMet	C175S	C175S + biuret	K142A	K142H
Spacegroup	C2	P2_1_2_1_2_1_	P2_1_2_1_2_1_	P22_1_2_1_	P22_1_2_1_
Cell (a x b x c)	135.7 x 101.0 x 65.6	74.2 x 86.9 x 343.0	73.7 x 87.3 x 341.7	62.1 x 122.2 x 136.1	62.1 x 122.7 x 135.7
Cell (α x β x γ)	90 x 91.8 x 90	90 x 90 x 90	90 x 90 x 90	90 x 90 x 90	90 x 90 x 90
Resolution (Å)	2.46	1.75	2.22	1.59	1.59
Completeness (%)	99.7 (98.0)	100 (100)	99.9 (98.6)	100 (100)	100 (100)
Rmerge %	0.279 (0.713)	0.090 (0.759)	0.159 (0.726)	0.183 (1.122)	0.071 (0.726)
Rpim %	0.091 (0.425)	0.056 (0.546)	0.093 (0.434)	0.074 (0.530)	0.045 (0.588)
Mean I/sigI	11.4 (2.7)	9.0 (1.1)	7.7 (2.5)	8.2 (1.6)	11.7 (1.6)
# unique reflections	32,094	223,984	110,156	139,549	139,814
Multiplicity	19.8 (7.4)	6.5 (5.2)	7.4 (7.1)	13.5 (10.4)	6.1 (4.3)
CC1/2	0.991 (0.817)	0.998 (0.847)	0.990 (0.652)	0.996 (0.665)	0.999 (0.627)
Anomalous completeness	99.5 (95.6)				
Anomalous multiplicity	10.0 (3.8)				
ΔAnom correlation between half sets	0.233 (inner = 0.795)				
# Se	24				
Wavelength (Å)	0.97919				
**Refinement**					
Resolution (Å)	45.0–2.46	44.5–1.75	42.9–2.22	50.0–1.59	50.0–1.59
No. Reflections	30,444	210,888	104,573	132,605	132,830
Rwork %	22.5	17.4	24.9	15.4	15.0
Rfree %	27.6	20.4	27.7	17.4	17.4
# atoms (total)	7,462	15,574	14,376	8,241	8,285
# waters	413	1445	409	879	959
# buffer/biuret/inhibitor atoms	0	0	49	112 / 22	56
Mean B value overall (Å^2^)	17.3	29.1	32.8	16.6	22.1
Mean B value protein (Å^2^)	17.9	29.3	33.5	16.3	21.8
Mean B value water (Å^2^)	10.7	36.8	26.3	26.8	32.4
Mean B value buffer/biuret/inhibitor (Å^2^)	NA	NA	24.1	20.6 / 30.2	31.3
r.m.s.d. bond lengths (Å^2^)	0.012	0.018	0.010	0.015	0.018
r.m.s.d. bond angles (°)	1.634	1.760	1.433	1.787	1.893
Ramachandran analysis (%) preferred/ outliers	97.8 / 0.1	97.9 / 0.1	98.2 / 0	97.9 / 0	97.9 / 0.1

### Molecular dynamics

A 500 ns molecular dynamics simulation was performed on the wild-type enzyme to investigate its substrate-active site interactions as well as the enzyme’s conformational plasticity. Beginning from the crystal structure (with ligand coordinates taken from the C175S mutant structure) the AmberTools package [45] was used to setup molecular dynamics simulations with the Amber2014SB force field for the protein degrees of freedom [46], the GAFF force field for the ligand degrees of freedom [47], and the OBC2 generalized-Born implicit solvent model [48,49]. Simulations were performed using the OpenMM simulation library [50] with a non-bonded cut-off of 1 nanometer, a salt concentration of 150 mM, and hydrogen bond lengths constrained. Equations of motion were integrated with a Langevin integrator (time step of 2 femtoseconds, a temperature of 300 Kelvin, and a collision frequency of 91/picosecond). After energy minimization, the system was equilibrated for 1 nanosecond, followed by 500 nanoseconds of simulation (collecting the positions of all atoms every 200 picoseconds).

The trajectory was subjected to further analysis *via* conformational clustering using the k-means algorithm with k = 20. These states were then assembled into a Markovian state model with a lag time of 40 nanoseconds using the PyEMMA python library [51]. The Caver algorithm [52,53] was used to identify a number of transiently forming tunnels linking the active sites of each monomer to the surface as well as the central cavity of the tetramer.

An additional simulation was performed using the same protocol to characterize the Cys175Ser mutant (which was observed to bind biuret but was non-catalytic)

### Enzyme assays

A glutamate dehydrogenase (GDH) coupled reaction was used to measure ammonia release in the biuret hydrolase (BiuH) dependent reactions. GDH catalyzes the NADH-dependent amination of α-ketoglutarate (S4 Fig). Ammonia production by BiuH was followed using the decrease of absorbance at 340 nm by UV spectrophotometry, which was due to the oxidation of NADH by GDH. 1.25 U of GDH was used per 250 μL reaction, the final concentrations of α-ketoglutarate and NADH were 3.5 mM and 0.2 mM, respectively.

Biuret hydrolase specific activity was obtained by using 22 nM of biuret hydrolase wild type or 0.22 μM of the variants and 5 mU/μL of GDH in presence of 1.2 mM of biuret in 25 mM potassium phosphate buffer pH 8.5, at 28 °C. Biuret hydrolase kinetic data were measured for the wild type and all the variants having a residual specific activity above 1% of the wild type enzymes, by using 22 nM of biuret hydrolase enzyme and either 2.9 μM or 0.9 μM of the variants, depending on their performance in presence of various concentrations of biuret ranging from 0–4 mM, using the GDH-coupled assay. All the kinetics constants were calculated using GraphPad Prism (GraphPad Software, San Diego, USA) fitting the rate data to the Michaelis-Menten equation:

$$\frac{d[P]}{dt}\;=\;\frac{V_{max}[S]}{K_{M}\;+\;[S]}$$

### Inhibition study

Inhibition of the BiuH’s activity was measured in the presence of *N*-carbamoyl-D,L-aspartic acid, using 22 nM of biuret hydrolase enzyme. A 100 mM stock solution of *N*-carbamoyl-D,L-aspartic acid was prepared in 50 mM HEPES buffer, pH 8.5. The IC_50_ was determined by measuring the catalytic rate of BiuH against 0.2 mM of biuret at 28 °C in 50 mM HEPES buffer, pH 8.5 in presence of increasing amount of inhibitor ranging from 0 to 20 mM (S5 Fig). *N*-carbamoyl-D,L-aspartic acid was shown to not limit GDH activity at 0–20 mM.

## Results and discussion

### Structure of biuret hydrolase

Although BiuH had been partially characterized previously [54], the structure had not been determined and the molecular detail of its biochemical activity had not been investigated. We obtained a number of X-ray structures of BiuH (Table 1), which allowed a more complete analysis of the enzyme. The native protein was purified (20 mg from 1 litre of culture, concentrated to 10 mg/mL) and crystallized after removal of the His-tag *in situ* using chymotrypsin, in neutral conditions containing medium or high molecular weight polyethylene glycols as precipitants. Proteolysis did not impact the activity of the purified enzyme (S6 Fig).

**Fig 2:**
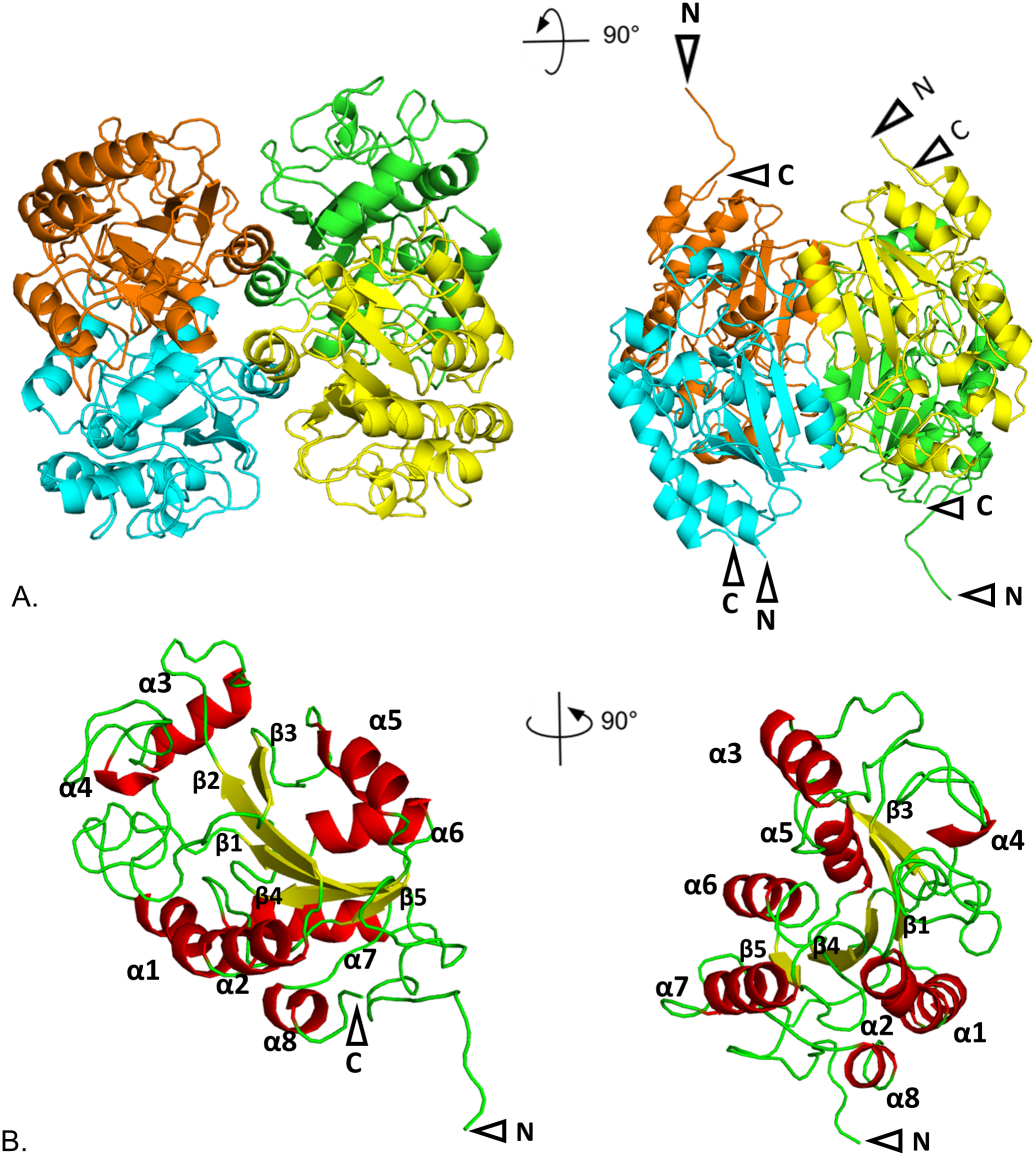
Structure of the biuret amidohydrolase enzyme. Cartoon representation of: A. BiuH tetrameric structure, each color represents a subunit; B. monomeric subunit of BiuH with alpha helices shown in red (numbered α1- α8), beta strands in yellow (numbered β1- β5) and loops in green. In every cases the N and/or C termini were visible, they are indicated by a black arrow. Figures 1, 3 and 6 were generated with PyMol [65].

The protein is a tetramer (consistent with SEC; S7 Fig); each monomer adopts a five-stranded parallel β-sheet which is surrounded by α-helices (Fig 2). Helices α2, α4 and α5 (residues 95–102, 178–186, and 202–213, respectively) make symmetric interactions to another protomer in the tetramer, forming a dimer, as part of the basis of the quaternary structure. Each of these dimer interfaces (A-D, B-C) cover an area of over 1800 Å^2^ whereas the interface between protomers not in the dimer (A-C, B-D) is significantly smaller (*ca*. 700 Å^2^). BiuH is similar to several other structures in the PDB ID: 3irv and 3uao (NicF; maleamate amidohydrolase; Uniprot: Q88FY5) [55–57] as examples (rmsd of Cα atoms of 1.3 to 1.6 Å over about 180 residues, sequence identities ranging from 26 to 29% identical). Excess densityassociated with Cys175, Cys114, Cys190 and Cys196 in the SeMet structure suggests that these residues have tendency to be oxidized. Depending on the structure and the chain, the loop containing residues 44–53 is at least somewhat disordered and has a higher B-factor than most of the rest of the protein. The average B factor for the chains where the loop is mobile (in more than one conformation) is about double that of the average B factor for both the whole of the protein chain and double what is seen for the same loop (44–53) in other chains where there is a single conformation.

### BiuH active site and catalytic mechanism

**Fig 3:**
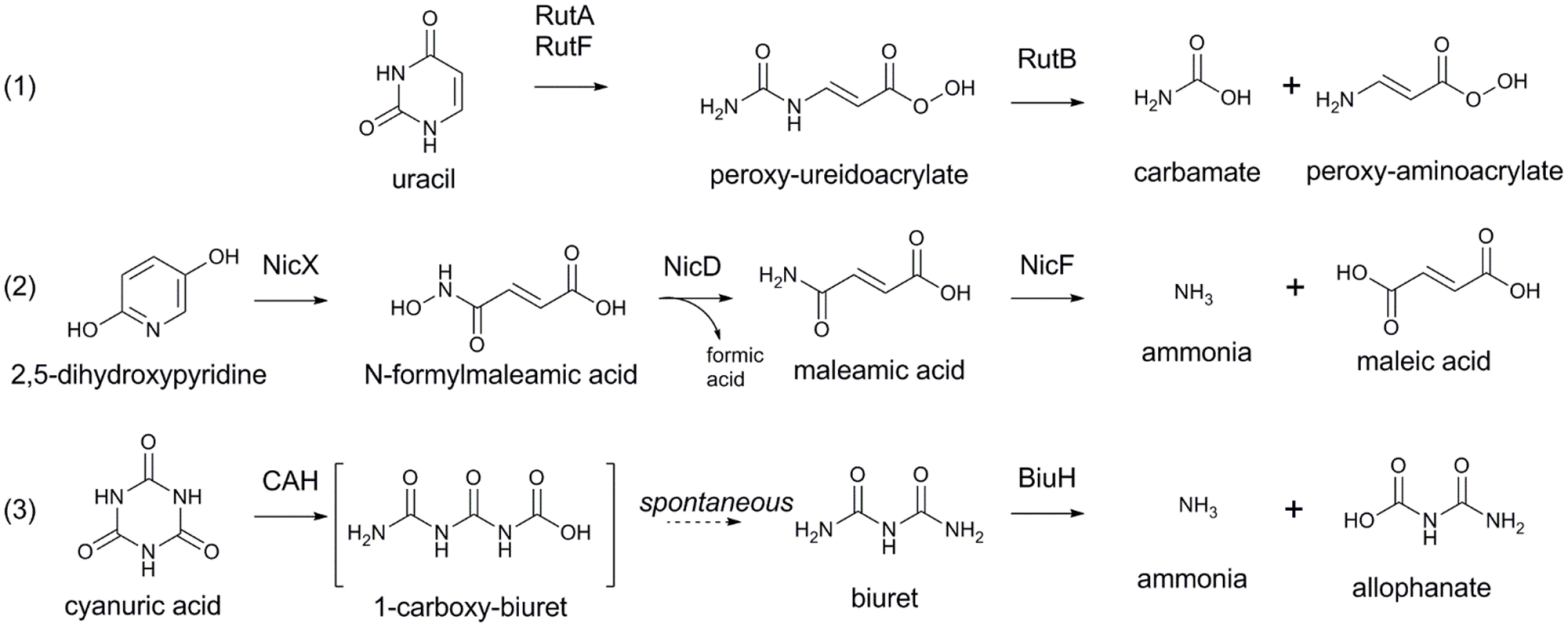
Reactions catalyzed by biuret amidohydrolase and homologs involved in heterocycle catabolism. 1) Ureidoacrylate peracid amidohydrolase (RutB) produces carbamate and peroxy-amino acrylate from peroxy-ureidoacrylate, which is produced by ring opening of uracil by RutA and RutF; 2) maleamate amidohydrolase (NicF) produces ammonia and maleic acid from maleamaic acid, produced by NicX and NicD from 2,5-dihydroxypyridine during nicotinic acid catabolism; and, 3) biuret amidohydrolase (BiuH) produces ammonia and allophanate from biuret during cyanuric acid catabolism.

BiuH belongs to the same family of proteins as RutB (ureidoacrylate peracid amidohydrolase; Uniprot: P75897) [58,59], PncA (nicotinamidase; Uniprot: P21369) [60–62] and NicF [55–57]. These enzymes are involved in the catabolism of heterocyclic compounds; PncA deaminates nicotinamide as part of the salvage pathway, RutB is required for the aerobic catabolism of pyrimidines and NicF is essential for nicotinic acid catabolism (Fig 2). PncA, NicF and RutB are all amidohydrolases; however, they catalyze slightly different reactions, with NicF and PncA perform analogous reactions producing ammonia and maleic acid (NicF) or nicotinic acid (PncA), while RutB produces aminoacrylate and carbamate. BiuH could potentially catalyze either reaction to produce either ammonia and allophanate or carbamate and urea. However, NMR studies with ^13^C labelled biuret provide strong evidence for the production of allophanate, rather than urea (Fig 3) [54].

Like RutB and NicF, BiuH contains no metal in the active site [58,59,63]. In this regard it differs from PncA, which contains a zinc that is con-ordinated by two histidine residues, an aspartate residue and two water atoms [64]. The bipyrimidal co-ordination of the active site zinc is completed by the nitrogen heteroatom of the nicotinamide ring, which positions the substrate for hydrolysis by the active site nucleophile (Cys159) [64]. This suggests that biuret hydrolase is more distantly related to PncA that it is to NicF or RutB.

A comparison of the sequences of biuret hydrolase with seven homologs (PDB ID: 3irv, 3kl2, 1nba, 3uao, 2wta, 3hu5, and 3r2j) indicated that nine amino acids are highly conserved in the active site of this enzyme family (Asp36, Gln38, Phe41, Lys142, Phe148, Thr151, Gly169, Cys175, Thr179). As Cys175 is the only conserved cysteine, it was probable that it was the active site nucleophile; indeed, substitution of Cys175 for isosteric serine abolished catalytic activity (without impacting protein folding; S2 Table). A structure of the inactive Cys175Ser variant with biuret in the active site was obtained (PDB ID: 6AZQ). Given the relatively minor change to the active site relative to that of the wild-type, this structure is a close approximation of the Michaelis complex before the nucleophilic attack on the substrate by active site nucleophile.

**Fig 4:**
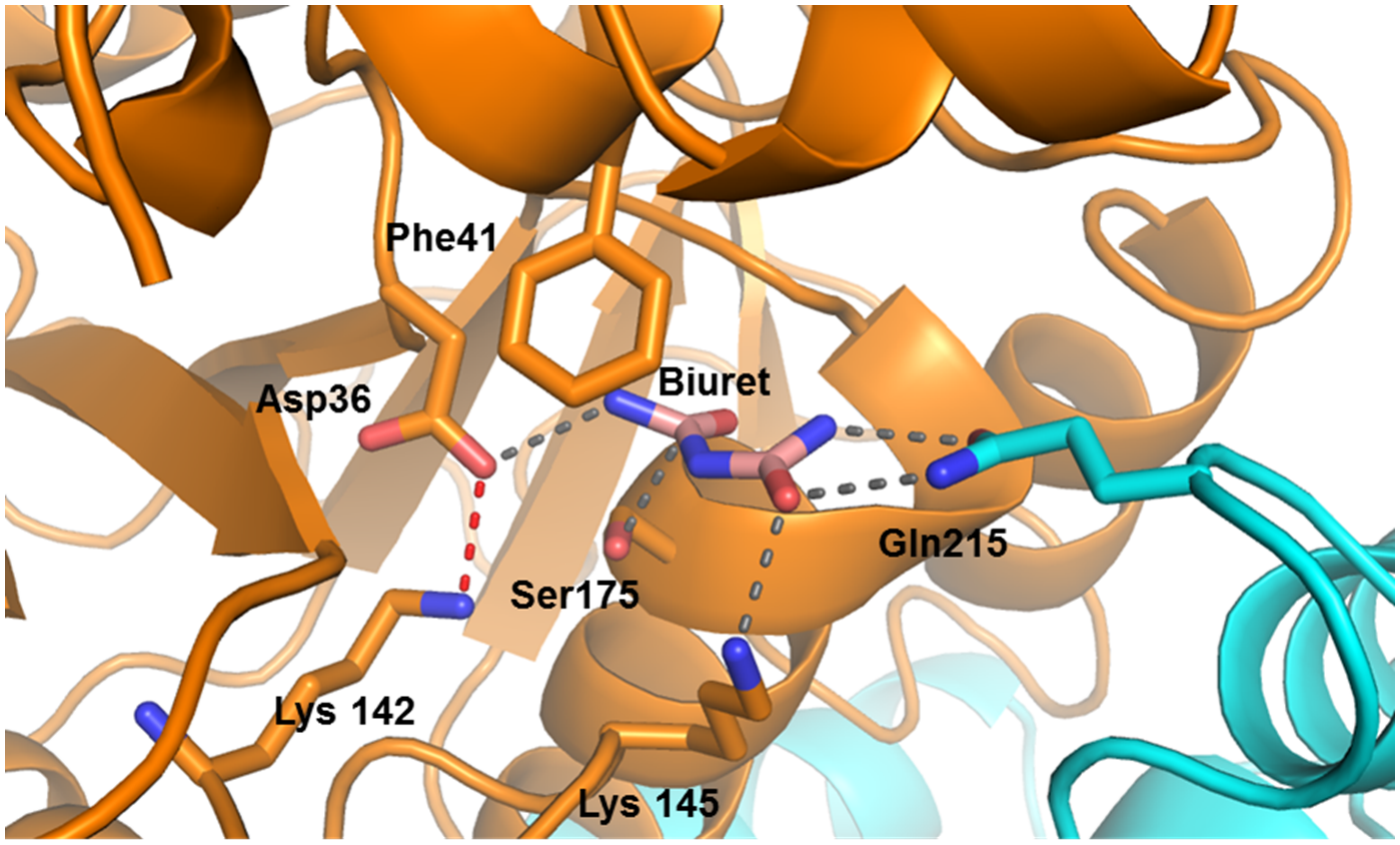
Cys175Ser BiuH variant showing biuret in the active site. BiuH is represented in cartoon style, with the exception of the active site amino acids; biuret is shown in pink, the hydrogen bonds between the residues and biuret are shown in grey, the hydrogen bonds between Asp36 and Lys142 in red; Gln215 is shown in cyan as it belongs to another enzyme subunit. The difference density map (Fo −Fc) of biuret in the active site is shown in S11 Fig.

In the Cys175Ser variant, biuret has an extensive hydrogen bond network with both sidechains and the protein backbone: Asp36, Lys145, Thr171, Ser175 and Gln215 have sidechain interactions; Ile170 and Thr171 contribute hydrogen bonds from backbone atoms. The active site Gln215 is provided by from the adjacent monomer of the dimer, which protrudes into the active site of its neighbouring monomer and binds the terminal amide that is distal from the nucleophile (Fig 4). The pocket is further constrained by Phe41, Tyr47 and Val174, with sidechain atoms of these residues between 3.2 and 3.9 Å away from the biuret molecule (Fig 4). The center of the Phe41 ring is about 3.0 Å from the N6 nitrogen, which is adjacent to the carbon presumed to be under attack by Cys175 in the wild-type enzyme. In this structure, Ser175, Asp36 and Lys142 are within hydrogen-bonding distance of one another; suggesting that, in addition to Cys175, Asp36 and Lys142 comprise the catalytic triad for BiuH. The amide oxygen closest to the nucleophile, which forms the oxyanion during catalysis, occupies a pocket that is formed by the main-chain nitrogens of Cys175 and Thr171. In NicF the oxyanion hole is formed by main-chain nitrogens of the nucleophilic cysteine (Cys150) and a threonine residue (Thr146) [63], and in PncA it is formed by main-chain nitrogens of a *cis*Ala (*cis*Ala155) and Phe (Phe158) [64]

**Fig 5:**
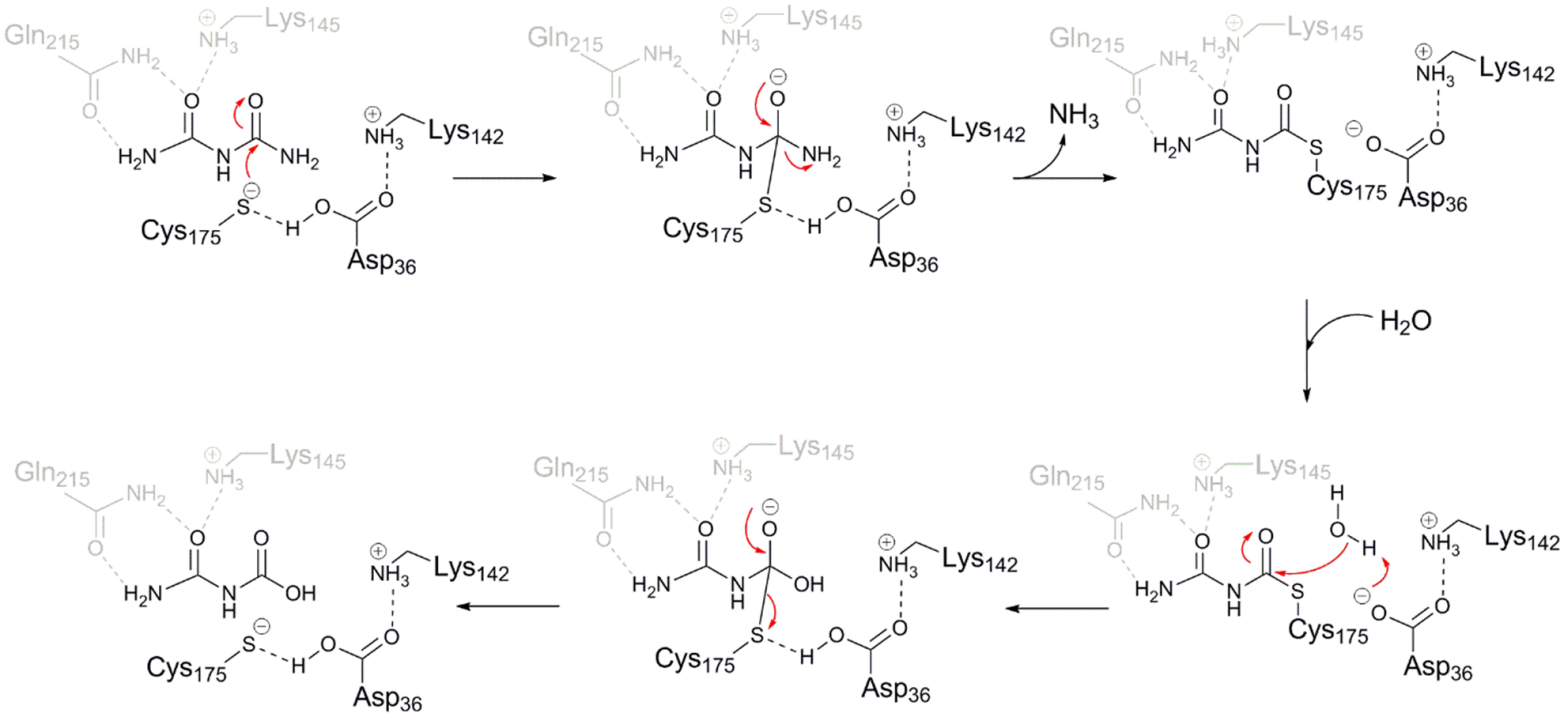
Suggested mechanism of the BiuH. Lys142 stabilizes Asp36 that will act as a general base and deprotonate Cys175, allowing Cys175 to perform a nucleophilic attack on the carbonyl end of biuret. Cys175 then binds to biuret forming a tetrahedral intermediate. Asp36 then acts as a general acid, leading to the collapse of the intermediate and the production of an ammonia and a thioester intermediate. Following the addition of a water molecule, Asp36 deprotonates the molecule of water leading to the hydrolysis of the thioester intermediate, forming a new tetrahedral intermediate. Finally, the enzyme is restored to its original state, releasing the allophanate product.

**Fig 6:**
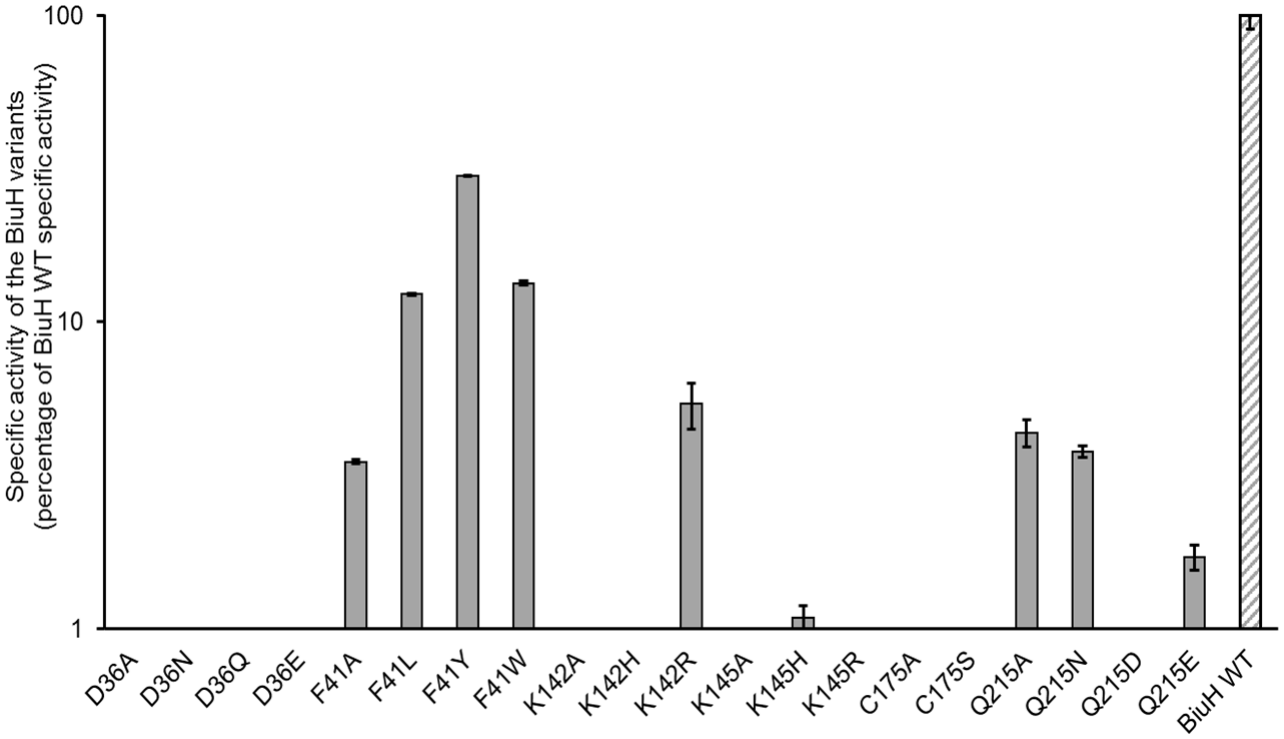
Specific activity of variant enzymes compared to the BiuH wild type enzyme. The specific activity is shown for each variant as percentage of the wild type BiuH specific activity, using 1.2 mM biuret as substrate (n = 3).

The catalytic mechanism of BiuH is therefore likely to be identical to that of other cysteine amidohydrolases (Fig 5) in which the active site nucleophile (Cys175) is deprotonated by a general base (Asp36). The function of Lys142 is likely to be the same as that of Lys117 in NicF, which serves to increase the acidity of Asp29 (the equivalent of BiuH Asp36) [55]. The nucleophile forms a tetrahedral covalent intermediate with the substrate, with the developing oxyanion stabilised by the main-chain nitrogens of Cys175 and Thr171. Thereafter ammonia is released and an acyl intermediate is formed between the substrate and enzyme. This intermediate is subsequently hydrolyzed to release the product (allophanate) and regenerate the active site for further catalysis (Fig 5). Mutagenesis of Asp36 and Lys142 support this mechanism (Fig 6, S2 Table): Asp36 cannot be substituted for other amino acids and Lys142 can only be replaced by an arginine residue (with a ~25-fold reduction in activity relative to the wild-type enzyme).

**Fig 7:**
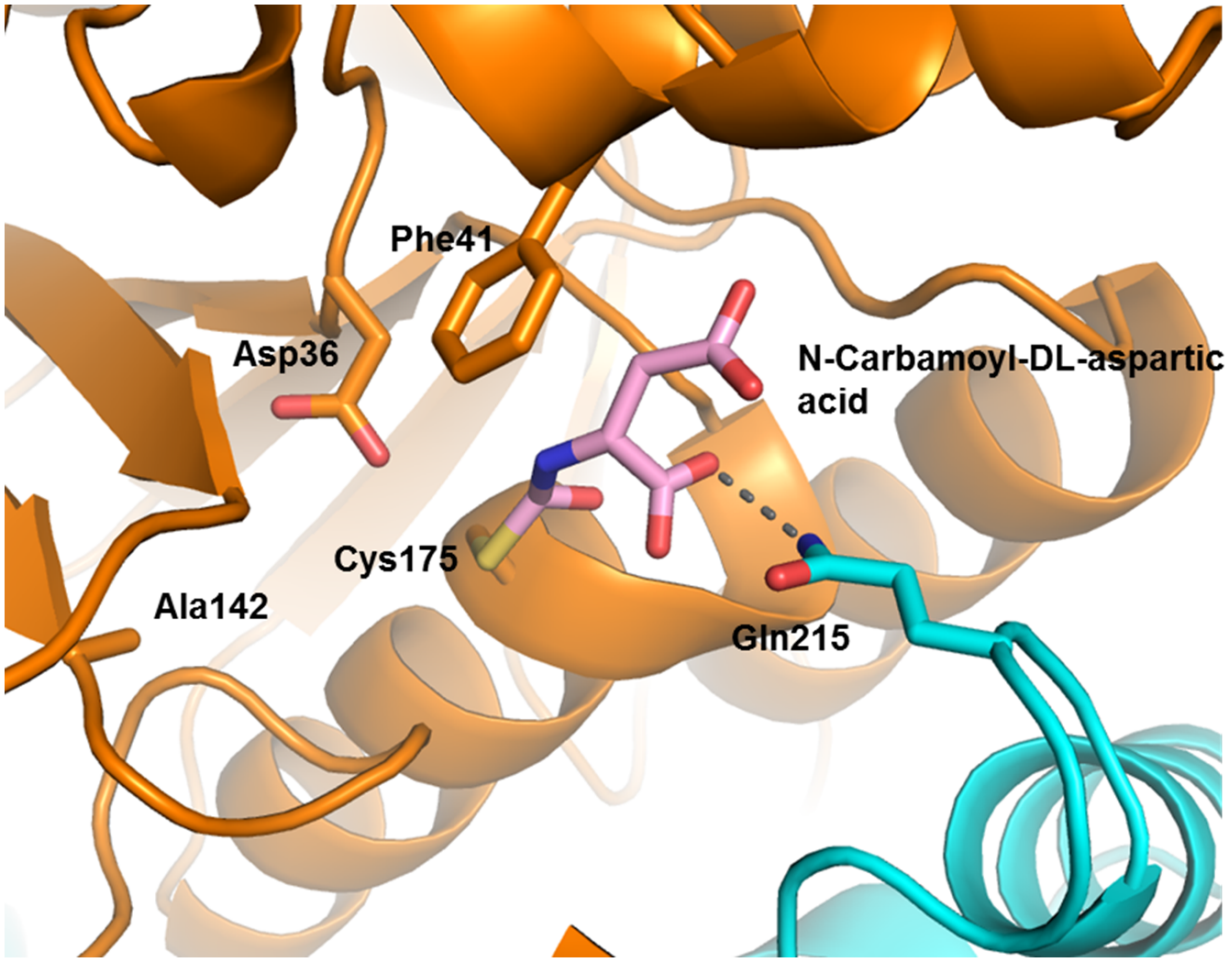
The active site of the Lys142Ala variant of BiuH showing Cys175 bound to the inhibitor *N*-carbamoyl-D,L-aspartic acid. BiuH is represented in cartoon style, with the exception of the active site amino acids; *N*-carbamoyl-D,L-aspartic acid is shown in pink, the hydrogen bond between the Gln215 and *N*-carbamoyl-D,L-aspartic acid are shown in grey, the hydrogen bonds between Asp36 and Ala142 in red; Gln215 is shown in cyan as it belongs to another enzyme subunit. The inhibition profile of BiuH with *N*-carbamoyl-D,L-aspartic acid is shown in S5 Fig and the difference density map (Fo −Fc) of the covalently bound inhibitor is shown in S11 Fig.

BiuH and its variants were treated with *N*-carbamoyl aspartic acid, which is a substrate analog previously demonstrated to inhibit BiuH activity [54]. The structure of the Lys142Ala variant was obtained after treatment with the inhibitor (PDB ID: 6AZS), and an acyl (thiocarbamate) covalent complex was captured between the deaminated inhibitor and Cys175 (Fig 7). This unequivocally demonstrates that Cys175 is the active site nucleophile, and also suggests a role for Lys142 in regenerating the active site after the formation of the covalent intermediate.

Variants were made that disrupted the hydrogen bonding interactions between the active site through hydrogen bonds with Gln215, Asp36, Lys145 and substrate. The purified variants were well folded, as shown by DSF, with T_m_ values from 41 °C (BiuH Asp36Glu) to 68 °C (BiuH Lys142His; PDB: 5BK6). The wild-type BiuH had an intermediate melting temperature of 56 °C. The Ser175 hydroxyl is less than 3 Å away from one of the two carbons in biuret, looking poised to attack. It should be noted that all heteroatoms (oxygen, nitrogen) in the biuret molecule hydrogen bond to the protein in an exquisite arrangement that makes the protein specific to its substrate. Steady state kinetics of the variants were obtained (Table 2). Substitution of Gln215 with Ala or Glu increased the *K*_M_ of BiuH for biuret by more than 25-fold (Table 2), consistent with the role of Gln215 in binding biuret. Interestingly, replacement of Gln215 with Asn slightly decreased the enzyme’s *K*_M_ for biuret, but reduced its *k*_cat_ by ~8-fold, presumably by modifying the orientation of the substrate in the active site relative to the nucleophilic thiolate of Cys175. Variants of Lys145 in which Lys was substituted for Ala, Arg or His were effectively inactive (albeit DSF showed them to be folded correctly; Table 2), suggesting that Lys145 may be essential for substrate binding (Lys145 has a 3.1 Å hydrogen bond directly to biuret). Phe41 is found in a mobile loop that appears to occlude the active site when not occupied by the substrate. Replacement of Phe41 with Ala, Leu, Tyr or Trp increased *K*_M_ and reduced *k*_cat_, suggesting a role in substrate binding and positioning, or in product egress.

**Table 2:** Steady state kinetic parameters of BiuH and its variants. (n = 5).

	*K*_M_ (μM)	*k*_cat_ (s^-1^)	*k*_cat_/*K*_M_ (s^-1^.M^-1^)
**BiuH WT**	79 ± 7	11.90 ± 0.300	149742
**Phe41Ala**	505 ± 23	0.14 ± 0.002	277
**Phe41Leu**	333 ± 15	0.07 ± 0.001	210
**Phe41Tyr**	749 ± 67	0.81 ± 0.026	1081
**Phe41Trp**	127 ± 8	0.26 ± 0.003	2047
**Gln215Ala**	2073 ± 100	0.74 ± 0.016	357
**Lys142Arg**	139 ± 8	0.07 ± 0.001	504
**Gln215Asn**	48 ± 2	0.19 ± 0.002	3958
**Gln215Glu**	2528 ± 197	0.29 ± 0.012	115

### Molecular dynamics

**Fig 8:**
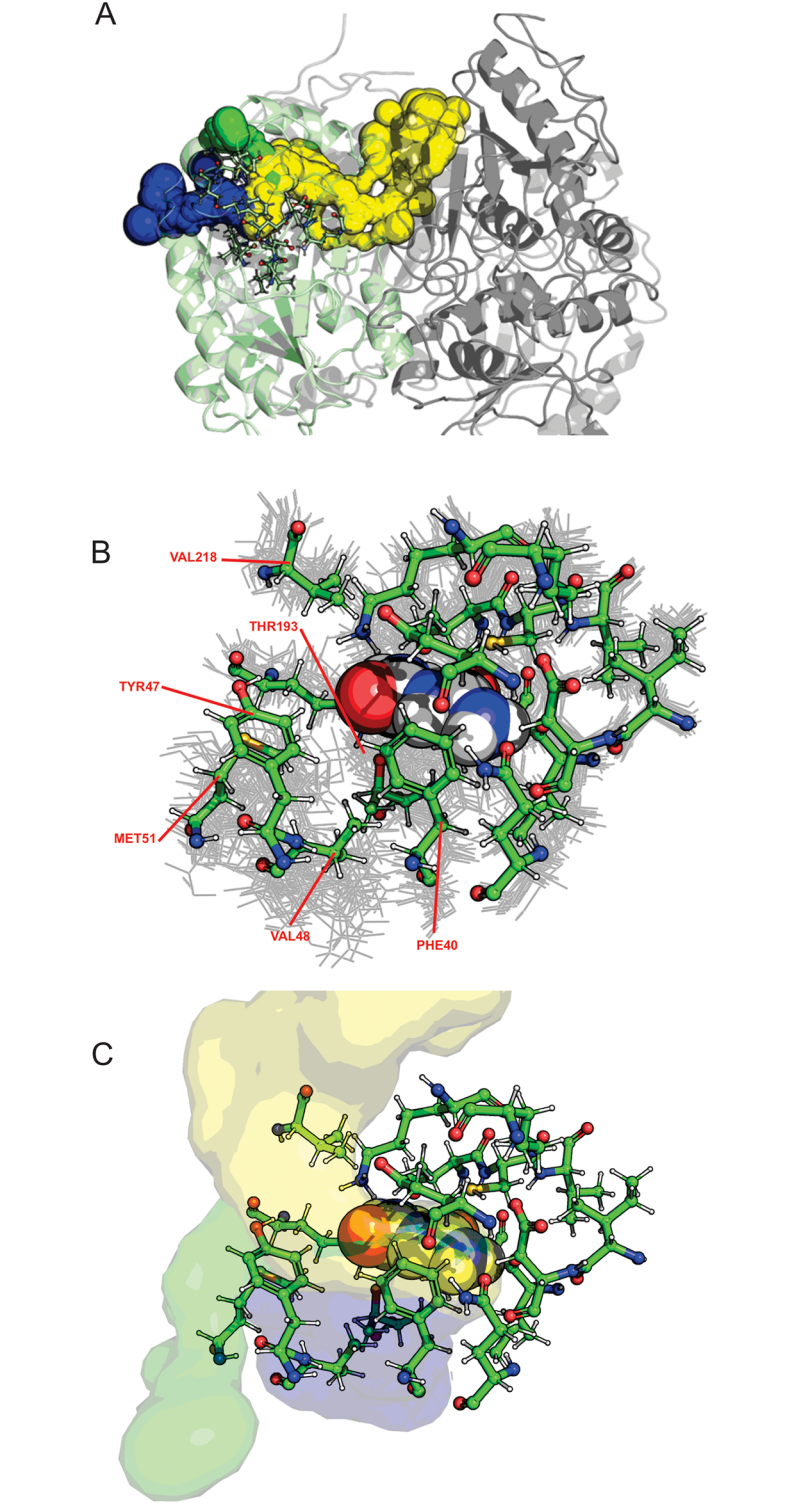
Solvent accessible channels in BiuH. Three solvent accessible channels emerging from the active site are shown in blue, green, and yellow. A) The blue and green tunnels quickly reach the surface of the monomer, while the yellow tunnel extends into the subunit interface and central cavity of the tetramer. The amino acids constituting the active site are shown as sticks, and the subunit that contained biuret is shown in pale green. B) The active site residues are shown as green sticks (for a representative structure of cluster 0) and as grey lines (for representative structures of other 19 clusters) and biuret is shown as Van der Waals spheres. The residues that are responsible for gating these tunnels are labeled in red C) The three dominant tunnels are shown on the right (green, yellow, and blue). Figure generated with Caver [52].

The binding of biuret to the wild type enzyme (PDB ID: 6AZO) was further investigated using molecular dynamics. A 500 ns trajectory indicated that biuret was held relatively tightly in the active site moving only slightly from the crystallographic conformation. Additionally, the catalytic residues Asp36 and Cys175 were rigid with Lys142 being somewhat more mobile. Examination of the conformations of the other active site residues revealed that a number of residues distal from the catalytic residues had either relatively high RMSD from the crystallographic conformation, or were shown to adopt two or three distinct rotomeric states (S8 and S9 Figs). From the Markovian state model there are four highly populated states,three more states with moderately low populations, and the remaining states had very low stationary probabilities (S9 Fig). Using these states as a guide, three putative tunnels were identified that extended from the surface of the protein into the active site, and were gated by the residues distal from the catalytic residues (Fig 8). The most persistent tunnel was gated by interactions with Val218 and Gln215 in the adjacent subunit while the smaller tunnels were gated by movement of Phe41, Tyr47, Val48, and Met51. Although further simulation is needed to verify this, it would seem that these secondary tunnels might serve to allow egress of ammonia and admission of water to enable the hydrolysis of the covalent intermediate.

Comparing simulations of the Cys175Ser mutant and the wild type revealed that Ser175 adopted a number of conformations that placed the nucleophilic oxygen an Ångstrom further from the appropriate atom in the substrate when compared to the wild-type (S10 Fig). This was exacerbated by the absence of productive hydrogen bonds with Glu84 and presence of non-productive hydrogen bonds with Thr171. Combined with the relatively higher p*K*_a_ of serine, these results help explain why the mutant tightly binds biuret, but does not catalyze its hydrolysis.

## Conclusion

Herein, we have described and characterized the structure and catalytic mechanism of BiuH, a cysteine hydrolase that hydrolytically deaminates biuret during cyanuric acid catabolism by *R*. *leguminosarum* bv. *viciae* 3841. BiuH is structurally and mechanistically different from the ‘canonical’ AtzE from *Pseudomonas* sp. strain ADP, which fulfils the same physiological role; i.e., it liberates a nitrogen atom from the triazine ring and allows further degradation to make all three nitrogen atoms bioavailable. Curiously, there is no equivalent of *atzF* in close proximity to the *atzD* and *BiuH* genes in *R*. *leguminosarum* bv. *viciae* 3841. Further study will be required to determine the metabolic fate of the allophanate produced by BiuH.

BiuH has an analogous function to enzymes in both pyridine and pyrimidine catabolism with which it shares sequence, structure and mechanistic similarity (i.e., NicF and RutB). This suggests that BiuH may have evolved from an ancestor with ureidoacrylate peracid amidohydrolase or maleamate amidohydrolase activity. Interestingly, although the gene cluster containing the cyanuric acid and biuret hydrolase in *Rhizobium leguminasorum* bv. *viciae* 3841 is the only such cluster characterized to date, similar (as yet uncharacterized) clusters are present in other bacteria: e.g., *Gordonia* sp. KTR9 (GenBank: AFR50980.1 and AFR50981.1), *Rhizobium leguminosarum* strain Vaf10 (GenBank:ANP91478.1 and ANP91508.1), *Rhizobium leguminosarum* bv. *trifolii* WSM1325 (GenBank:ACS61202.1 and ACS61203.1), *Rhizobium tropici* CIAT 899 (GenBank:AGB75492.1 and AGB75491.1) and *Agrobacterium vitis* S4 (GenBank:ACM38740.1 and ACM38741.1). This may suggest that BiuH homologs play a more important role than anticipated in the environmental fate of *s*-triazine compounds.

## Supporting information

S1 Fig Protein sequence of the biuret hydrolase protein from *Rhizobium leguminosarum* bv. *viciae* 3841. EMBL database accession no. AM236084.1, containing the 6xhis-tag and the thrombin cleavage sites added through the cloning (in red).
(PDF)

S2 Fig Meltdown output for BiuH. (PDF)

S3 Fig Melting temperature of BiuH and its variants. The melting temperature (Tm) was measured by differential scanning fluorimetry in °C (n = 3–16 depending on the variants).
(PDF)

S4 Fig Biuret hydrolase (BiuH) activity assays. A glutamate dehydrogenase (GDH) coupled reaction was used to measure ammonium release in the assays of the biuret hydrolase WT and its variants.
(PDF)

S5 Fig Percentage of BiuH activity in function of the *N*-Carbamoyl-D,L-aspartic acid inhibitor concentration (mM). The activity of BiuH was measured with 0.2 mM biuret and the GDH-coupled assay in the presence of increasing amounts of inhibitor (n = 6); *N*-Carbamoyl-DL-aspartic acid structure is shown on the top right corner.
(PDF)

S6 Fig Steady state kinetic parameters of BiuH with/without his-tag and comparison to published data. (PDF)

S7 Fig Size exclusion chromatographies from BiuH or its variants. Number 8 represents the elution time for a protein having the size of an octamer of BiuH (red), 4 represents the size of a tetramer of BiuH (blue), 2 the elution time for a protein having the size of a dimer of BiuH (black) and 1 the elution time expected for a protein of the size of the BiuH monomer. The size exclusion from the K145A/H/R variants seem to present a slight shift towards the dimeric size when compared to the other variants.
(PDF)

S8 Fig Root mean square distance for BiuH active site and biuret during molecular dynamics simulation. Top: Root mean square distance from the crystallographic conformation for active site residues and biuret over the course of a 500 ns molecular dynamics simulation. Bottom: Distributions of root mean square distances from the crystallographic conformation for active side residues during a 500 ns molecular dynamics simulation.
(PDF)

S9 Fig Conformational clustering of active site residues throughout a 500ns molecular dynamics simulation. States were assigned by clustering the Cartesian coordinates of the active site residues and biuret using the k-means algorithm (k = 20). Top: Discrete trajectory showing the conformational state of the active site throughout the course of the simulation. Bottom: Network diagram showing Markovian state model (lag time = 40 ns) of the conformational transitions between clustered states. The area of each node is proportional to the equilibrium probability of the state while the thickness of the arrows is proportional to the transition probability.
(PDF)

S10 Fig Comparison of the biuret–enzyme interactions observed during molecular dynamics simulations for the WT enzyme (blue) and the C175S mutant (orange). The top row of plots shows the time trace of the number of hydrogen bonds between the active site residues and biuret, a histogram of these hydrogen bonds, and a bar chart highlighting differences in hydrogen bonding between WT and mutant. On the bottom, the distance between the nucleophile and the electrophilic carbon of biuret is shown as a function of time on the left, and distributions on the right.
(PDF)

S11 Fig Difference density map (Fo −Fc) shown around biuret and the N-carbamoyl-D,L-aspartic acid inhibitor. A. Active site of the Cys175Ser BiuH variant shown in cartoon, with the active site amino acids and biuret shown in stick and the difference density map (Fo −Fc) in green mesh. B. Active site of the Lys142Ala variant of BiuH showing Cys175 bound to the inhibitor N-carbamoyl-D,L-aspartic acid and the difference density map (Fo −Fc), with the active site amino acids and the inhibitor shown in stick and the difference density map (Fo −Fc) in green mesh.
(PDF)

S1 Table Mutagenic primers used for introducing point mutation in BiuH’s sequence by overlapping PCR. In bold are the base pairs causing the mutation.
(PDF)

S2 Table Specific activity of BiuH and its variants. The specific activity was measured in presence of 1.2 mM of biuret (n = 3) in μmoles.sec-1.mg enzyme-1, Tm: melting temperature measured by differential scanning fluorimetry in °C (n = 3–16 depending on the variants).
(PDF)

S3 Table Crystallisation conditions. (PDF)

## References

[1] Wackett LP (2009) Questioning our perceptions about evolution of biodegradative enzymes. Current Opinion in Microbiology 12: 244–251. doi: 10.1016/j.mib.2009.05.001 19477677 10.1016/j.mib.2009.05.001

[2] Udikovic-Kolic N, Scott C, Martin-Laurent F (2012) Evolution of atrazine-degrading capabilities in the environment. Applied Microbiology and Biotechnology 96: 1175–1189. doi: 10.1007/s00253-012-4495-0 23076592 10.1007/s00253-012-4495-0

[3] Wackett LP (2004) Evolution of new enzymes and pathways: Soil microbes adapt to s-triazine herbicides In: Gan JJ, Zhu PC, Aust SD, Lemley AT, editors. Pesticide Decontamination and Detoxification. Washington: Amer Chemical Soc pp. 37–48.

[4] Wackett LP (2005) Biodegradation of *s*-triazine herbicides. Abstracts of Papers of the American Chemical Society 230: U2153–U2153.

[5] Wackett LP (2001) Evolution and global distribution on triazine-catabolic enzymes. Biochemistry 40: 8636–8636. 10.1021/bi011293r 11669610

[6] Russell RJ, Scott C, Jackson CJ, Pandey R, Pandey G, Taylor MC, et al (2011) The evolution of new enzyme function: lessons from xenobiotic metabolizing bacteria versus insecticide-resistant insects. Evolutionary Applications 4: 225–248. doi: 10.1111/j.1752-4571.2010.00175.x 25567970 10.1111/j.1752-4571.2010.00175.x PMC3352558

[7] Peat TS, Newman J, Balotra S, Lucent D, Warden AC, Scott C (2015) The structure of the hexameric atrazine chlorohydrolase AtzA. Acta Crystallographica Section D-Biological Crystallography 71: 710–720. 10.1107/S1399004715000619 PMC4356373 25760618

[8] Boundy-Mills KL, de Souza ML, Mandelbaum RT, Wackett LP, Sadowsky MJ (1997) The *atzB* gene of *Pseudomonas* sp strain ADP encodes the second enzyme of a novel atrazine degradation pathway. Applied and Environmental Microbiology 63: 916–923. 9055410 10.1128/aem.63.3.916-923.1997 PMC168384

[9] Balotra S, Warden AC, Newman J, Briggs LJ, Scott C, Peat TS (2015) X-Ray structure and mutagenesis studies of the N-isopropylammelide isopropylaminohydrolase, AtzC. PLoS One 10. 10.1371/journal.pone.0137700 PMC4577212 26390431

[10] Peat TS, Balotra S, Wilding M, French NG, Briggs LJ, Panjikar S, et al (2013) Cyanuric acid hydrolase: evolutionary innovation by structural concatenation. Molecular Microbiology 88: 1149–1163. doi: 10.1111/mmi.12249 23651355 10.1111/mmi.12249 PMC3758960

[11] Seffernick JL, Erickson JS, Cameron SM, Cho S, Dodge AG, Richman JE, et al (2012) Defining sequence space and reaction products within the cyanuric acid hydrolase (AtzD)/barbiturase protein family. Journal of Bacteriology 194: 4579–4588. doi: 10.1128/JB.00791-12 22730121 10.1128/JB.00791-12 PMC3415516

[12] Fruchey I, Shapir N, Sadowsky MJ, Wackett LP (2003) On the origins of cyanuric acid hydrolase: Purification, substrates, and prevalence of AtzD from *Pseudomonas* sp strain ADP. Applied and Environmental Microbiology 69: 3653–3657. doi: 10.1128/AEM.69.6.3653-3657.2003 12788776 10.1128/AEM.69.6.3653-3657.2003 PMC161465

[13] Cameron SM, Sadowsky MJ, Wackett LP (2011) Novel biuret hydrolase reveals presence of food toxicant, cyanuric acid. Abstracts of Papers of the American Chemical Society 241: 1.

[14] Martinez B, Tomkins J, Wackett LP, Wing R, Sadowsky MJ (2001) Complete nucleotide sequence and organization of the atrazine catabolic plasmid pADP-1 from *Pseudomonas* sp. strain ADP. Journal of Bacteriology 183: 5684–5697. doi: 10.1128/JB.183.19.5684-5697.2001 11544232 10.1128/JB.183.19.5684-5697.2001 PMC95461

[15] Balotra S, Newman J, Cowieson NP, French NG, Campbell PM, Briggs LJ, et al (2015) X-Ray structure of the amidase domain of AtzF, the allophanate hydrolase from the cyanuric acid-mineralizing multienzyme complex. Applied and Environmental Microbiology 81: 470–480. doi: 10.1128/AEM.02783-14 25362066 10.1128/AEM.02783-14 PMC4277574

[16] Shapir N, Cheng G, Sadowsky MJ, Wackett LP (2006) Purification and characterization of TrzF: Biuret hydrolysis by allophanate hydrolase supports growth. Applied and Environmental Microbiology 72: 2491–2495. doi: 10.1128/AEM.72.4.2491-2495.2006 16597948 10.1128/AEM.72.4.2491-2495.2006 PMC1449057

[17] Cheng G, Shapir N, Sadowsky MJ, Wackett LP (2005) Allophanate hydrolase, not urease, functions in bacterial cyanuric acid metabolism. Applied and Environmental Microbiology 71: 4437–4445. doi: 10.1128/AEM.71.8.4437-4445.2005 16085834 10.1128/AEM.71.8.4437-4445.2005 PMC1183272

[18] Shapir N, Sadowsky MJ, Wackett LP (2005) Purification and characterization of allophanate hydrolase (AtzF) from *Pseudomonas* sp strain ADP. Journal of Bacteriology 187: 3731–3738. doi: 10.1128/JB.187.11.3731-3738.2005 15901697 10.1128/JB.187.11.3731-3738.2005 PMC1112067

[19] Balotra S, Warden AC, Newman J, Briggs LJ, Scott C, Peat TS (2015) X-Ray Structure and Mutagenesis Studies of the N-Isopropylammelide Isopropylaminohydrolase, AtzC. Plos One 10: 15. 10.1371/journal.pone.0137700 PMC4577212 26390431

[20] Peat TS, Balotra S, Wilding M, Hartley CJ, Newman J, Scott C (2017) High-resolution X-ray structures of two functionally distinct members of the cyclic amide hydrolase family of Toblerone fold enzymes. Applied and Environmental Microbiology 83: 13. 10.1128/AEM.03365-16 PMC5394311 28235873

[21] Shapir N, Pedersen C, Gil O, Strong L, Seffernick J, Sadowsky MJ, et al (2006) TrzN from *Arthrobacter aurescens* TC1 is a zinc amidohydrolase. Journal of Bacteriology 188: 5859–5864. doi: 10.1128/JB.00517-06 16885454 10.1128/JB.00517-06 PMC1540083

[22] Shapir N, Rosendahl C, Johnson G, Andreina M, Sadowsky MJ, Wackett LP (2005) Substrate specificity and colorimetric assay for recombinant TrzN derived from *Arthrobacter aurescens* TC1. Applied and Environmental Microbiology 71: 2214–2220. doi: 10.1128/AEM.71.5.2214-2220.2005 15870302 10.1128/AEM.71.5.2214-2220.2005 PMC1087567

[23] Sugrue E, Carr PD, Scott C, Jackson CJ (2016) Active site desolvation and thermostability trade-offs in the evolution of catalytically diverse triazine hydrolases. Biochemistry 55: 6304–6313. doi: 10.1021/acs.biochem.6b00731 27768291 10.1021/acs.biochem.6b00731

[24] Jutzi K, Cook AM, Hutter R (1981) The degradative pathway of the *s-*triazine melamine. Experientia 37: 1231–1232. 10.1042/bj2080679 PMC1154018 6762212

[25] Cook AM, Hutter R (1981) *s*-Triazines as nitrogen sources for bacteria. Journal of Agricultural and Food Chemistry 29: 1135–1143.

[26] Seffernick JL, de Souza ML, Sadowsky MJ, Wackett LP (2001) Melamine deaminase and atrazine chlorohydrolase: 98 percent identical but functionally different. Journal of Bacteriology 183: 2405–2410. doi: 10.1128/JB.183.8.2405-2410.2001 11274097 10.1128/JB.183.8.2405-2410.2001 PMC95154

[27] Young JPW, Crossman LC, Johnston AWB, Thomson NR, Ghazoui ZF, Hull KH, et al (2006) The genome of *Rhizobium leguminosarum* has recognizable core and accessory components. Genome Biology 7. 10.1186/gb-2006-7-4-r34 PMC1557990 16640791

[28] Jackson CJ, Coppin CW, Carr PD, Aleksandrov A, Wilding M, Sugrue E, et al (2014) 300-Fold increase in production of the Zn^2+^-dependent dechlorinase TrzN in soluble form *via* apoenzyme stabilization. Applied and Environmental Microbiology 80: 4003–4011. doi: 10.1128/AEM.00916-14 24771025 10.1128/AEM.00916-14 PMC4054219

[29] Scott C, Jackson CJ, Coppin CW, Mourant RG, Hilton ME, Sutherland TD, et al (2009) Catalytic improvement and evolution of atrazine chlorohydrolase. Applied and Environmental Microbiology 75: 2184–2191. doi: 10.1128/AEM.02634-08 19201959 10.1128/AEM.02634-08 PMC2663207

[30] Scott C, Lewis SE, Milla R, Taylor MC, Rodgers AJW, Dumsday G, et al (2010) A free-enzyme catalyst for the bioremediation of environmental atrazine contamination. Journal of Environmental Management 91: 2075–2078. doi: 10.1016/j.jenvman.2010.05.007 20570036 10.1016/j.jenvman.2010.05.007

[31] Radian A, Aukema KG, Aksan A, Wackett LP (2015) Silica gel for enhanced activity and hypochlorite protection of cyanuric acid hydrolase in recombinant *Escherichia coli*. Mbio 6: 11. 10.1128/mBio.01477-15 PMC4631802 26530383

[32] Yeom S, Mutlu BR, Aksan A, Wackett LP (2015) Bacterial cyanuric acid hydrolase for water treatment. Applied and Environmental Microbiology 81: 6660–6668. doi: 10.1128/AEM.02175-15 26187963 10.1128/AEM.02175-15 PMC4561718

[33] Mac Mahon S, Begley TH, Diachenko GW, Stromgren SA (2012) A liquid chromatography-tandem mass spectrometry method for the detection of economically motivated adulteration in protein-containing foods. Journal of Chromatography A 1220: 101–107. doi: 10.1016/j.chroma.2011.11.066 22197251 10.1016/j.chroma.2011.11.066

[34] Achor DS, Albrigo LG (2005) Biuret toxicity symptoms in citrus leaves mimics cell senescence rather than nutritional deficiency chlorosis. Journal of the American Society for Horticultural Science 130: 667–673.

[35] Shen RC (1959) Fertilizer contaminants -rate of biuret formation from urea. Journal of Agricultural and Food Chemistry 7: 762–763.

[36] Ho SN, Hunt HD, Horton RM, Pullen JK, Pease LR (1989) Site-directed mutagenesis by overlap extension by the polymerase chain-reaction. Gene 77: 51–59. 2744487 10.1016/0378-1119(89)90358-2

[37] Doublie S (1997) Preparation of selenomethionyl proteins for phase determination. Macromolecular Crystallography, Pt A 276: 523–530. 9048379

[38] Seabrook SA, Newman J (2013) High-throughput thermal scanning for protein stability: Making a good technique more robust. Acs Combinatorial Science 15: 387–392. doi: 10.1021/co400013v 23710551 10.1021/co400013v

[39] Rosa N, Ristic M, Seabrook SA, Lovell D, Lucent D, Newman J (2015) Meltdown: A tool to help in the interpretation of thermal melt curves acquired by differential scanning fluorimetry. Journal of Biomolecular Screening 20: 898–905. doi: 10.1177/1087057115584059 25918038 10.1177/1087057115584059

[40] Skubak P, Pannu NS (2013) Automatic protein structure solution from weak X-ray data. Nature Communications 4: 6. 10.1038/ncomms3777 PMC3868232 24231803

[41] McCoy AJ (2007) Solving structures of protein complexes by molecular replacement with Phaser. Acta Crystallographica Section D-Biological Crystallography 63: 32–41. 10.1107/S0907444906045975 PMC2483468 17164524

[42] Emsley P, Cowtan K (2004) Coot: model-building tools for molecular graphics. Acta Crystallographica Section D-Biological Crystallography 60: 2126–2132. 10.1107/S0907444904019158 15572765

[43] Murshudov GN, Skubak P, Lebedev AA, Pannu NS, Steiner RA, Nicholls RA, et al (2011) REFMAC5 for the refinement of macromolecular crystal structures. Acta Crystallographica Section D-Biological Crystallography 67: 355–367. 10.1107/S0907444911001314 PMC3069751 21460454

[44] Adams PD, Afonine PV, Bunkoczi G, Chen VB, Davis IW, Echols N, et al (2010) PHENIX: a comprehensive Python-based system for macromolecular structure solution. Acta Crystallographica Section D-Biological Crystallography 66: 213–221. 10.1107/S0907444909052925 PMC2815670 20124702

[45] D.A. Case DA, Cerutti DS, Cheatham III TE, Darden TA, Duke RE, Giese TJ, et al. (2017) AMBER 2017. San Francisco: University of California.

[46] Maier JA, Martinez C, Kasavajhala K, Wickstrom L, Hauser KE, Simmerling C (2015) ff14SB: Improving the accuracy of protein side chain and backbone parameters from ff99SB. Journal of Chemical Theory and Computation 11: 3696–3713. doi: 10.1021/acs.jctc.5b00255 26574453 10.1021/acs.jctc.5b00255 PMC4821407

[47] Wang JM, Wolf RM, Caldwell JW, Kollman PA, Case DA (2004) Development and testing of a general AMBER force field. Journal of Computational Chemistry 25: 1157–1174. doi: 10.1002/jcc.20035 15116359 10.1002/jcc.20035

[48] Onufriev A, Bashford D, Case DA (2004) Exploring protein native states and large-scale conformational changes with a modified generalized born model. Proteins-Structure Function and Bioinformatics 55: 383–394. 10.1002/prot.20033 15048829

[49] Nguyen H, Roe DR, Simmerling C (2013) Improved generalized Born solvent model parameters for protein simulations. Journal of Chemical Theory and Computation 9: 2020–2034. doi: 10.1021/ct3010485 25788871 10.1021/ct3010485 PMC4361090

[50] Eastman P, Friedrichs MS, Chodera JD, Radmer RJ, Bruns CM, Ku JP, et al (2013) OpenMM 4: A reusable, extensible, hardware independent library for high performance molecular simulation. Journal of Chemical Theory and Computation 9: 461–469. doi: 10.1021/ct300857j 23316124 10.1021/ct300857j PMC3539733

[51] Scherer MK, Trendelkamp-Schroer B, Paul F, Perez-Hernandez G, Hoffmann M, Plattner N, et al (2015) PyEMMA 2: A software package for estimation, validation, and analysis of Markov models. Journal of Chemical Theory and Computation 11: 5525–5542. doi: 10.1021/acs.jctc.5b00743 26574340 10.1021/acs.jctc.5b00743

[52] Pavelka A, Sebestova E, Kozlikova B, Brezovsky J, Sochor J, Damborsky J (2016) CAVER: Algorithms for analyzing dynamics of tunnels in macromolecules. IEEE-ACM Transactions on Computational Biology and Bioinformatics 13: 505–517. doi: 10.1109/TCBB.2015.2459680 27295634 10.1109/TCBB.2015.2459680

[53] Pavelka A, Chovancova E, Damborsky J (2009) HotSpot Wizard: a web server for identification of hot spots in protein engineering. Nucleic Acids Research 37: W376–W383. doi: 10.1093/nar/gkp410 19465397 10.1093/nar/gkp410 PMC2703904

[54] Cameron SM, Durchschein K, Richman JE, Sadowsky MJ, Wackett LP (2011) New family of biuret hydrolases involved in *s*-triazine ring metabolism. ACS Catalysis 1: 1075–1082. 10.1021/cs200295n PMC3166513 21897878

[55] Kincaid VA, Sullivan ED, Klein RD, Noel JW, Rowlett RS, Snidert MJ (2012) Structure and catalytic mechanism of nicotinate (vitamin B3) degradative enzyme maleamate amidohydrolase from *Bordetella bronchiseptica* RB50. Biochemistry 51: 545–554. doi: 10.1021/bi201347n 22214383 10.1021/bi201347n

[56] Jimenez JI, Juarez JF, Garcia JL, Diaz E (2011) A finely tuned regulatory circuit of the nicotinic acid degradation pathway in *Pseudomonas putida*. Environmental Microbiology 13: 1718–1732. doi: 10.1111/j.1462-2920.2011.02471.x 21450002 10.1111/j.1462-2920.2011.02471.x

[57] Jimenez JI, Canales A, Jimenez-Barbero J, Ginalski K, Rychlewski L, Garcia JL, et al (2008) Deciphering the genetic determinants for aerobic nicotinic acid degradation: The *nic* cluster from *Pseudomonas putida* KT2440. Proceedings of the National Academy of Sciences of the United States of America 105: 11329–11334. doi: 10.1073/pnas.0802273105 18678916 10.1073/pnas.0802273105 PMC2516282

[58] Parales RE, Ingraham JL (2010) The surprising Rut pathway: an unexpected way to derive nitrogen from pyrimidines. Journal of Bacteriology 192: 4086–4088. doi: 10.1128/JB.00573-10 20562306 10.1128/JB.00573-10 PMC2916418

[59] Kim KS, Pelton JG, Inwood WB, Andersen U, Kustu S, Wemmer DE (2010) The Rut pathway for pyrimidine degradation: Novel chemistry and toxicity problems. Journal of Bacteriology 192: 4089–4102. doi: 10.1128/JB.00201-10 20400551 10.1128/JB.00201-10 PMC2916427

[60] Gazzaniga F, Stebbins R, Chang SZ, McPeek MA, Brenner C (2009) Microbial NAD metabolism: Lessons from comparative genomics. Microbiology and Molecular Biology Reviews 73: 529-+. doi: 10.1128/MMBR.00042-08 19721089 10.1128/MMBR.00042-08 PMC2738131

[61] French JB, Cen Y, Sauve AA, Ealick SE (2010) High-resolution crystal structures of *Streptococcus pneumoniae* nicotinamidase with trapped intermediates provide insights into the catalytic mechanism and inhibition by aldehydes. Biochemistry 49: 8803–8812. doi: 10.1021/bi1012436 20853856 10.1021/bi1012436 PMC3006156

[62] Frothingham R, MeekerOconnell WA, Talbot EAS, George JW, Kreuzer KN (1996) Identification, cloning, and expression of the *Escherichia coli* pyrazinamidase and nicotinamidase gene, *pncA*. Antimicrobial Agents and Chemotherapy 40: 1426–1431. 8726014 10.1128/aac.40.6.1426 PMC163344

[63] Kincaid VA, Sullivan ED, Klein RD, Noel JW, Rowlett RS, Snidert MJ (2012) Structure and Catalytic Mechanism of Nicotinate (Vitamin B-3) Degradative Enzyme Maleamate Amidohydrolase from Bordetella bronchiseptica RB50. Biochemistry 51: 545–554. doi: 10.1021/bi201347n 22214383 10.1021/bi201347n

[64] Fyfe PK, Rao VA, Zemla A, Cameron S, Hunter WN (2009) Specificity and mechanism of *Acinetobacter baumanii* nicotinamidase: implications for activation of the front-Line tuberculosis drug pyrazinamide. Angewandte Chemie-International Edition 48: 9176–9179. doi: 10.1002/anie.200903407 19859929 10.1002/anie.200903407 PMC3465764

[65] Schrodinger, LLC (2015) The PyMOL Molecular Graphics System, Version 1.8.

