# Supplementary material for: Structural and biochemical characterization of the biuret hydrolase (BiuH) from the cyanuric acid catabolism pathway of Rhizobium leguminasorum bv. viciae 3841
Source: PLoS One. 2018 Feb 9;13(2):e0192736. doi: 10.1371/journal.pone.0192736 (PMC5806882; doi:10.1371/journal.pone.0192736)
Supplement: S1 Fig — EMBL database accession no. AM236084.1, containing the 6xhis-tag and the thrombin cleavage sites added through the cloning (in red). (PDF) [file pone.0192736.s001.pdf]

S1 Figure: Protein sequence of the biuret hydrolase protein from *Rhizobium leguminosarum* bv. *viciae* 3841. EMBL database accession no. AM236084.1, containing the 6xhis-tag and the thrombin cleavage sites added through the cloning (in red).

MGSSHHHHHHSSGLVPRGSHMPWMDAMVETNRHFIDADPYPWPYNGALRPDNTALIIIDMQT

DFCGKGGYVDHMGYDLSLVQAPIEPIKRVLAAMRAKGYHIIHTREGHRPDLADLPANKRWRS

QRIGAGIGDPGPCGRILTRGEPGWDIIPELYPIEGETIIDKPGKGSFCATDLELVLNQKRIE

NIILTGITTDVCVSTTMREANDRGYECLLLEDCCGATDYGNHLAAIKMVKMQGGVFGSVSNS

AALVEALP
