# Supplementary material for: Structural and biochemical characterization of the biuret hydrolase (BiuH) from the cyanuric acid catabolism pathway of Rhizobium leguminasorum bv. viciae 3841
Source: PLoS One. 2018 Feb 9;13(2):e0192736. doi: 10.1371/journal.pone.0192736 (PMC5806882; doi:10.1371/journal.pone.0192736)
Supplement: S2 Fig — (PDF) [file pone.0192736.s002.pdf]

MELTDOWN v2.0.0 Melt Curve Analysis

cysteinehydrolase-tris-thrombin_Buffer-sc...

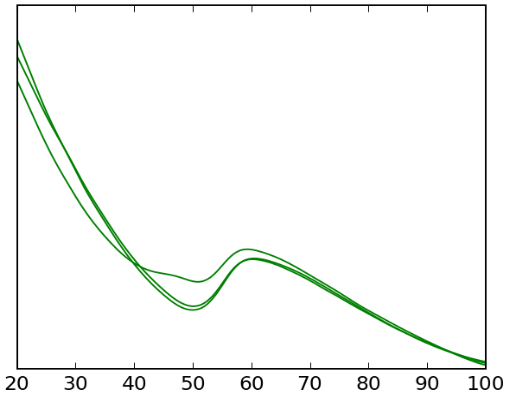

Full interpretation of the results requires you to look at the individual melt curves.

77% of curves were used in Tm estimations

Average estimation of error is **0.3 C**

Protein as supplied is **well behaved**

Protein as supplied: Tm = 54.92(+/-0.09)

Lysozyme Control: Passed
No Dye Control: Passed
No Protein Control: Passed

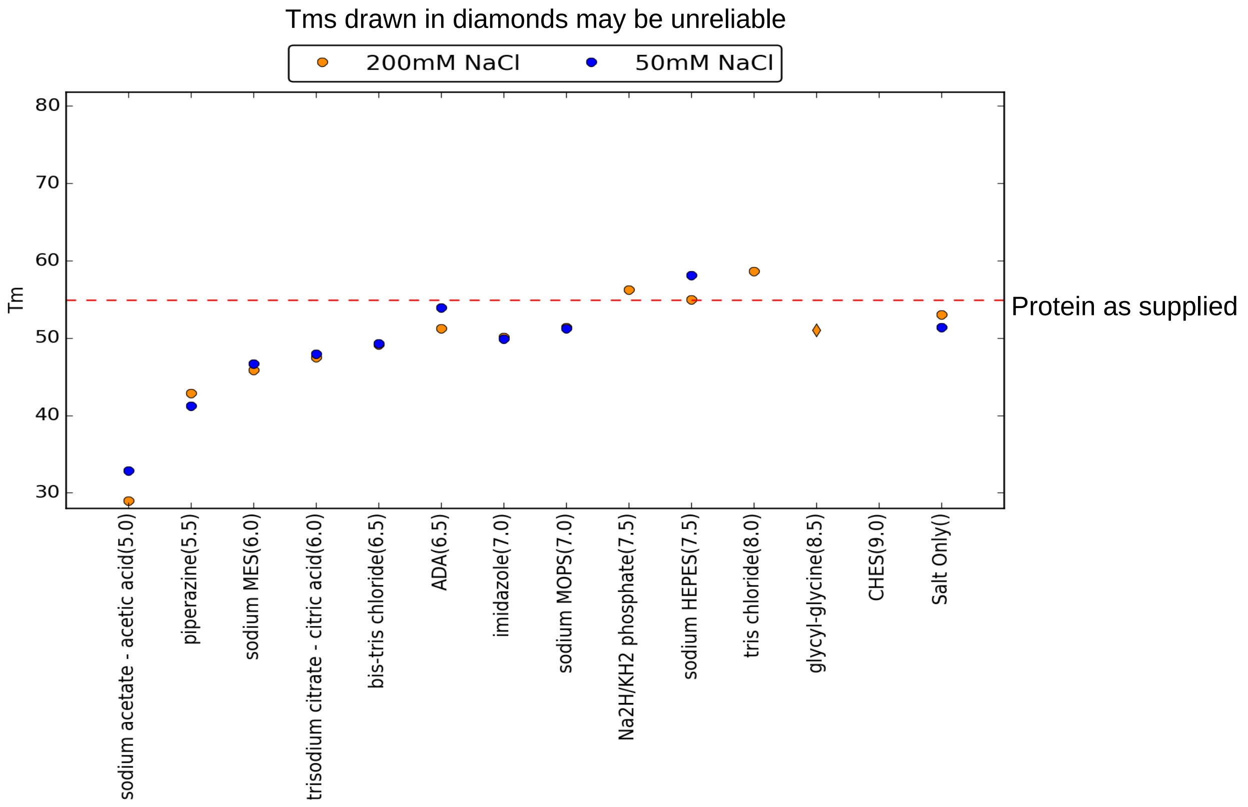

Highest Tm = 58.68 +/- 0.25
(tris chloride / 200mM NaCl)

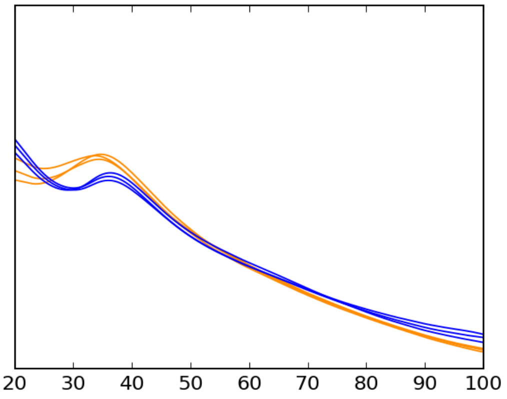

sodium acetate - acetic acid (5.0)

Grouped by	Tm
200mM NaCl	29.0 (+/-0.1)
50mM NaCl	32.83 (+/-0.07)

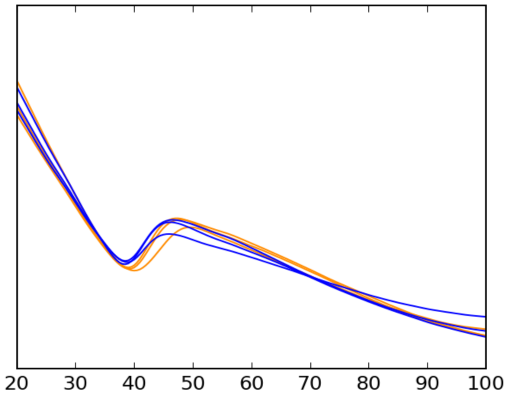

piperazine (5.5)

Grouped by	Tm	Adjusted pH at Tm
200mM NaCl	42.87 (+/-0.85)	5.08
50mM NaCl	41.27 (+/-0.09)	5.11

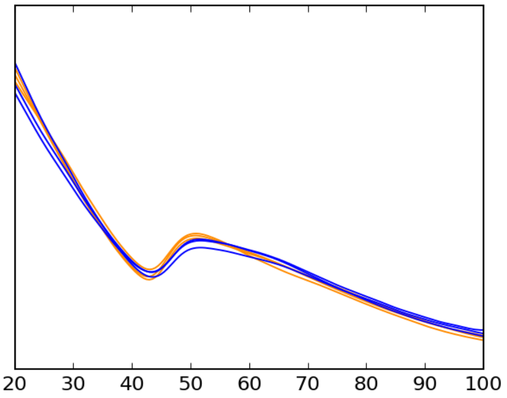

sodium MES (6.0)

Grouped by	Tm	Adjusted pH at Tm
200mM NaCl	45.85 (+/-0.13)	5.8
50mM NaCl	46.68 (+/-0.07)	5.79

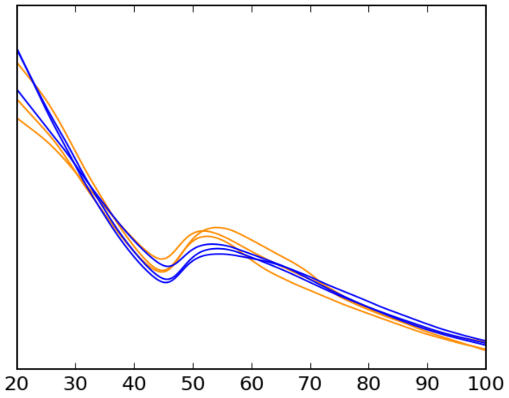

trisodium citrate - citric acid (6.0)

Grouped by	Tm	Adjusted pH at Tm
200mM NaCl	47.55 (+/-0.21)	6.11
50mM NaCl	47.98 (+/-0.13)	6.11

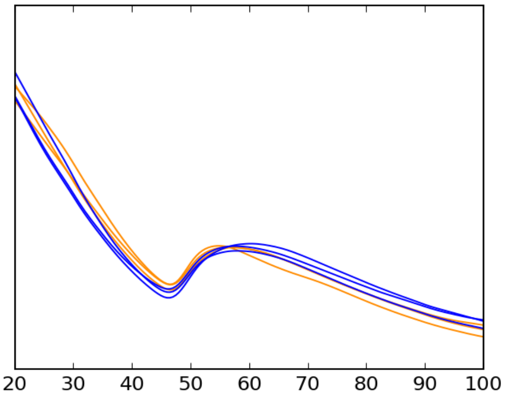

bis-tris chloride (6.5)

Grouped by	Tm	Adjusted pH at Tm
200mM NaCl	49.17 (+/-0.06)	6.12
50mM NaCl	49.34 (+/-0.06)	6.12

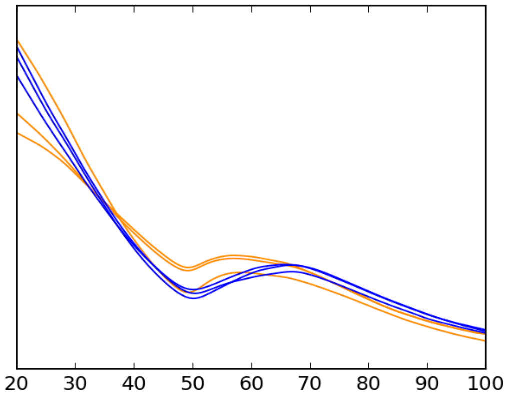

ADA (6.5)

Grouped by	Tm	Adjusted pH at Tm
200mM NaCl	51.27 (+/-0.12)	6.39
50mM NaCl	53.98 (+/-0.56)	6.38

Monotonic, saturated, in the noise, and outlier curves are dotted, and excluded from Tm calculations
Curves drawn with dashed lines have unreliable Tm estimates

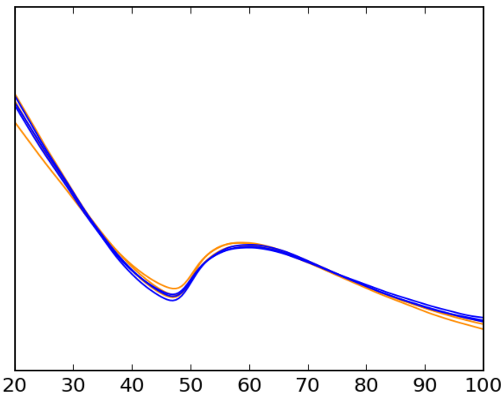

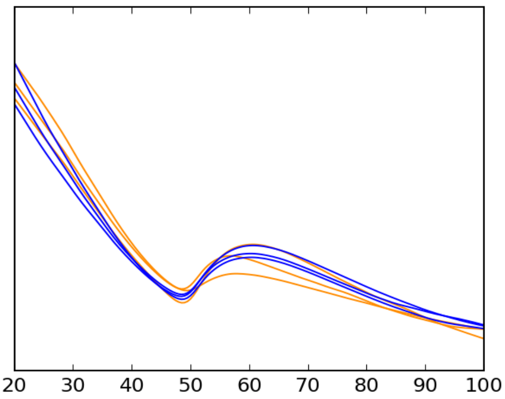

imidazole (7.0)

Grouped by	Tm	Adjusted pH at Tm
200mM NaCl	50.12 (+/-0.08)	6.49
50mM NaCl	49.94 (+/-0.03)	6.49

sodium MOPS (7.0)

Grouped by	Tm	Adjusted pH at Tm
200mM NaCl	51.43 (+/-0.26)	6.74
50mM NaCl	51.25 (+/-0.04)	6.74

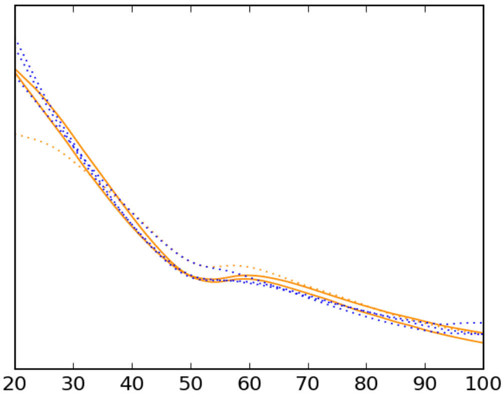

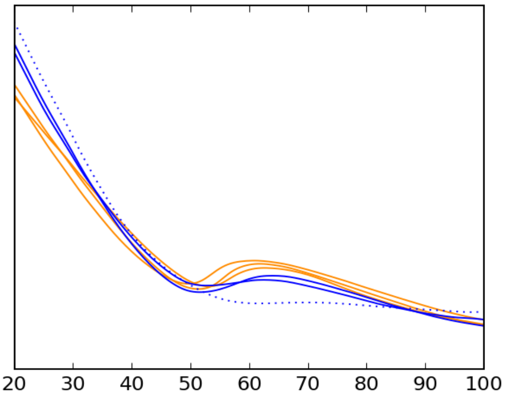

Na2H/KH2 phosphate (7.5)

Grouped by	Tm	Adjusted pH at Tm
200mM NaCl	56.27 (+/-0.01)	7.7
50mM NaCl	None	

sodium HEPES (7.5)

Grouped by	Tm	Adjusted pH at Tm
200mM NaCl	55.01 (+/-1.24)	7.29
50mM NaCl	58.1 (+/-0.35)	7.27

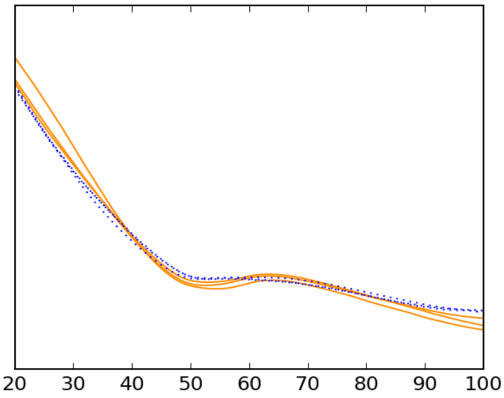

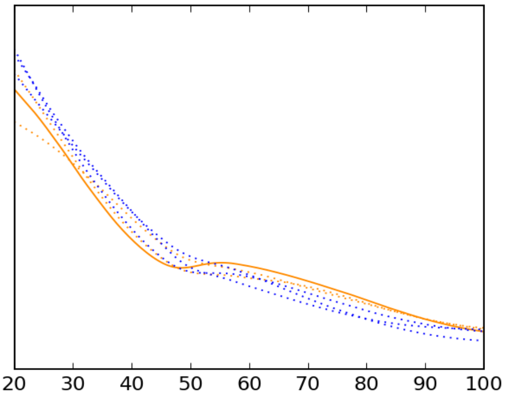

tris chloride (8.0)

Grouped by	Tm	Adjusted pH at Tm
200mM NaCl	58.68 (+/-0.25)	7.22
50mM NaCl	None	

glycyl-glycine (8.5)

Grouped by	Tm	Adjusted pH at Tm
200mM NaCl	51.04	7.98
50mM NaCl	None	

Monotonic, saturated, in the noise, and outlier curves are dotted, and excluded from Tm calculations
Curves drawn with dashed lines have unreliable Tm estimates

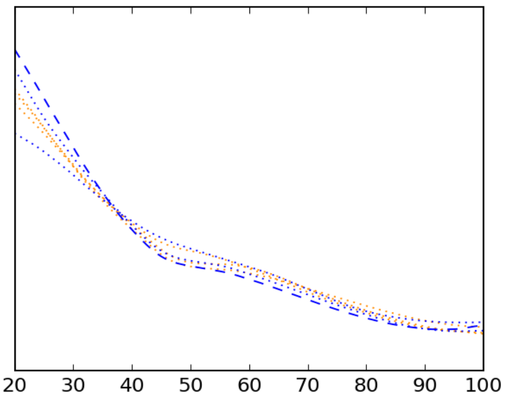

CHES (9.0)

Grouped by	Tm
200mM NaCl	None
50mM NaCl	None

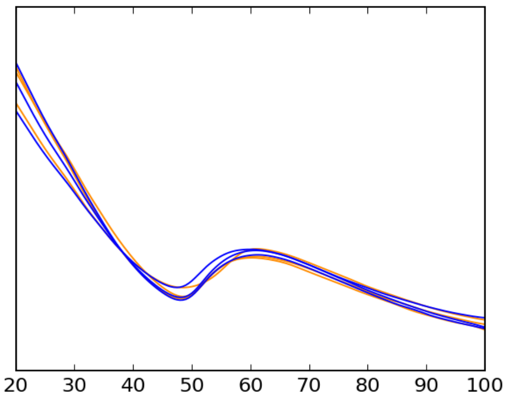

Salt Only ()

Grouped by	Tm
200mM NaCl	53.06 (+/-1.76)
50mM NaCl	51.4 (+/-0.29)

Monotonic, saturated, in the noise, and outlier curves are dotted, and excluded from Tm calculations
Curves drawn with dashed lines have unreliable Tm estimates
