# Supplementary material for: Structural and biochemical characterization of the biuret hydrolase (BiuH) from the cyanuric acid catabolism pathway of Rhizobium leguminasorum bv. viciae 3841
Source: PLoS One. 2018 Feb 9;13(2):e0192736. doi: 10.1371/journal.pone.0192736 (PMC5806882; doi:10.1371/journal.pone.0192736)

S3 Figure: Melting temperature of BiuH and its variants. The melting temperature (T_m) was measured by differential scanning fluorimetry in °C (n=3-16 depending on the variants).

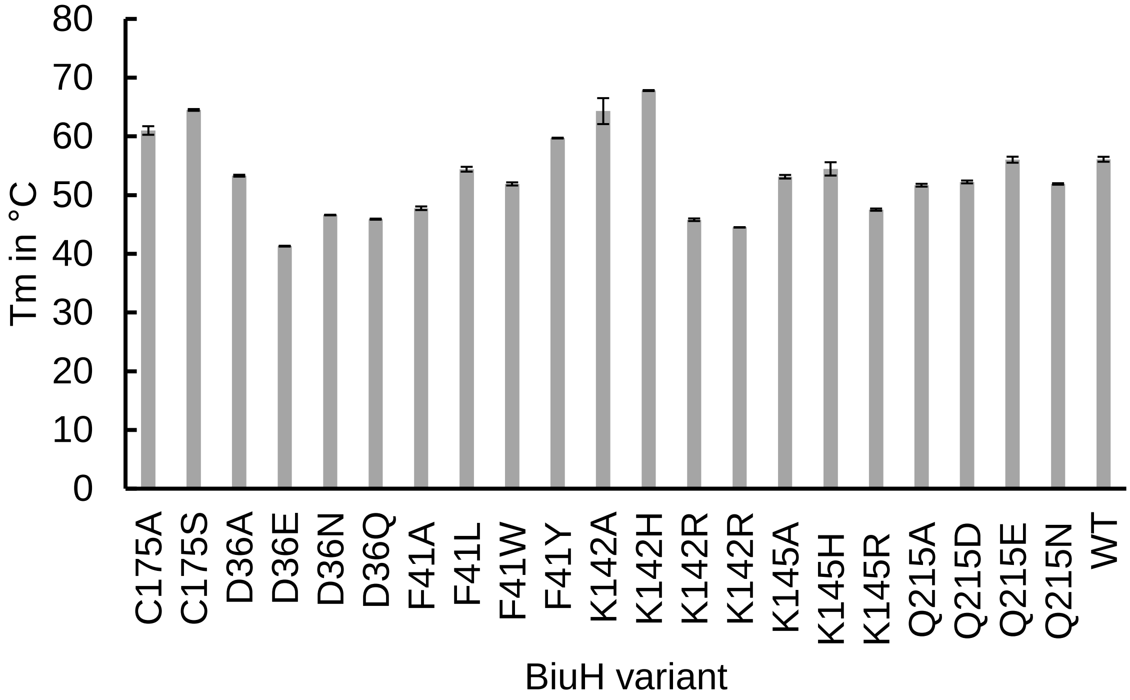

Supplement: S3 Fig — The melting temperature (Tm) was measured by differential scanning fluorimetry in °C (n = 3–16 depending on the variants). (PDF) [file pone.0192736.s003.pdf]
