# Supplementary material for: Structural and biochemical characterization of the biuret hydrolase (BiuH) from the cyanuric acid catabolism pathway of Rhizobium leguminasorum bv. viciae 3841
Source: PLoS One. 2018 Feb 9;13(2):e0192736. doi: 10.1371/journal.pone.0192736 (PMC5806882; doi:10.1371/journal.pone.0192736)

S4 Fig: Biuret hydrolase (BiuH) activity assays. A glutamate dehydrogenase (GDH) coupled reaction was used to measure ammonium release in the assays of the biuret hydrolase WT and its variants.

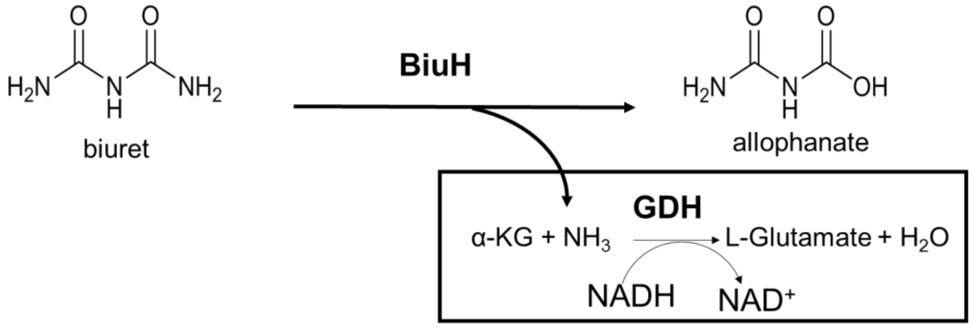

Supplement: S4 Fig — A glutamate dehydrogenase (GDH) coupled reaction was used to measure ammonium release in the assays of the biuret hydrolase WT and its variants. (PDF) [file pone.0192736.s004.pdf]
