# Supplementary material for: Structural and biochemical characterization of the biuret hydrolase (BiuH) from the cyanuric acid catabolism pathway of Rhizobium leguminasorum bv. viciae 3841
Source: PLoS One. 2018 Feb 9;13(2):e0192736. doi: 10.1371/journal.pone.0192736 (PMC5806882; doi:10.1371/journal.pone.0192736)

**S5 Fig: Percentage of BiuH activity in function of the *N*-Carbamoyl-D,L-aspartic acid inhibitor concentration (mM).** The activity of BiuH was measured with 0.2 mM biuret and the GDH-coupled assay in the presence of increasing amounts of inhibitor (n=6); *N*-Carbamoyl-DL-aspartic acid structure is shown on the top right corner.

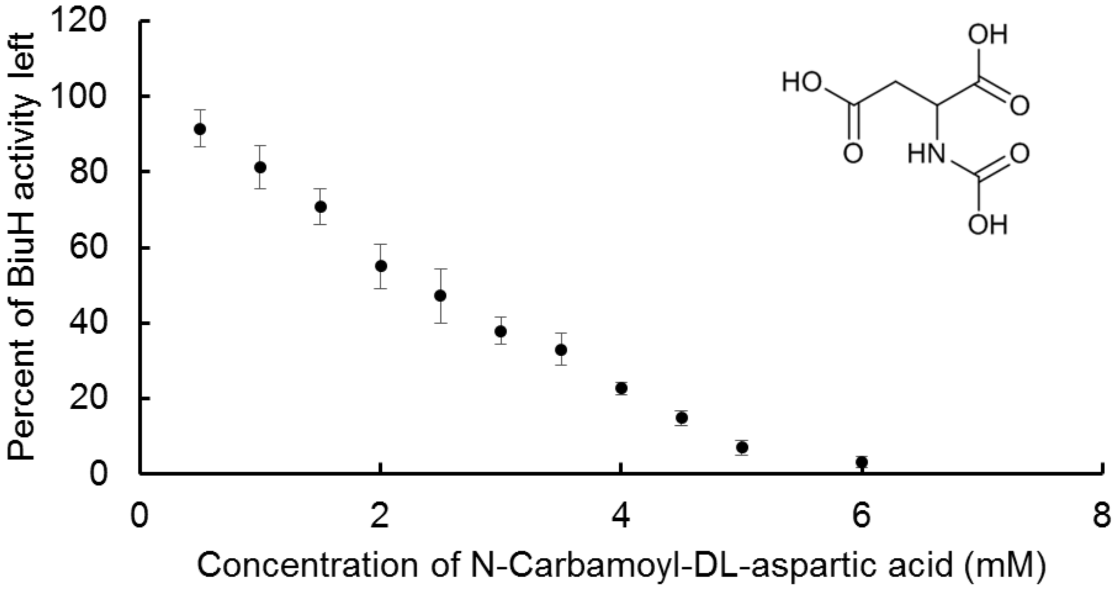

Supplement: S5 Fig — The activity of BiuH was measured with 0.2 mM biuret and the GDH-coupled assay in the presence of increasing amounts of inhibitor (n = 6); N-Carbamoyl-DL-aspartic acid structure is shown on the top right corner. (PDF) [file pone.0192736.s005.pdf]
