# Supplementary material for: Structural and biochemical characterization of the biuret hydrolase (BiuH) from the cyanuric acid catabolism pathway of Rhizobium leguminasorum bv. viciae 3841
Source: PLoS One. 2018 Feb 9;13(2):e0192736. doi: 10.1371/journal.pone.0192736 (PMC5806882; doi:10.1371/journal.pone.0192736)
Supplement: S6 Fig — (PDF) [file pone.0192736.s006.pdf]

S6 Fig. Steady state kinetic parameters of BiuH with/without his-tag and comparison to published data.

	K_M (μM)	k_{cat} (s^{-1})	k_{cat}/K_M ($s^{-1}.M^{-1}$)
**BiuH WT with his-tag**	79 ± 7	11.9 ± 0.3	1.5 x 10^5
**BiuH WT without his-tag**	74 ± 2	8.8 ± 0.1	1.2 x 10^5
**BiuH WT, as published by Cameron *et al* 2011**	23 ± 4	4.0 ± 0.2	1.7 x 10^5
