# Supplementary material for: Structural and biochemical characterization of the biuret hydrolase (BiuH) from the cyanuric acid catabolism pathway of Rhizobium leguminasorum bv. viciae 3841
Source: PLoS One. 2018 Feb 9;13(2):e0192736. doi: 10.1371/journal.pone.0192736 (PMC5806882; doi:10.1371/journal.pone.0192736)
Supplement: S7 Fig — Number 8 represents the elution time for a protein having the size of an octamer of BiuH (red), 4 represents the size of a tetramer of BiuH (blue), 2 the elution time for a protein having the size of a dimer of BiuH (black) and 1 the elution time expected for a protein of the size of the BiuH monomer. The size exclusion from the K145A/H/R variants seem to present a slight shift towards the dimeric size when compared to the other variants. (PDF) [file pone.0192736.s007.pdf]

Size Exclusion chromatographies from BiuH or its variants. Number 8 represents the elution time for a protein having the size of an octamer of BiuH (red), 4 represents the size of a tetramer of BiuH (blue), 2 the elution time for a protein having the size of a dimer of BiuH (black) and 1 the elution time expected for a protein of the size of the BiuH monomer. The size exclusion from the K145A/H/R variants seem to present a slight shift towards the dimeric size when compared to the other variants.

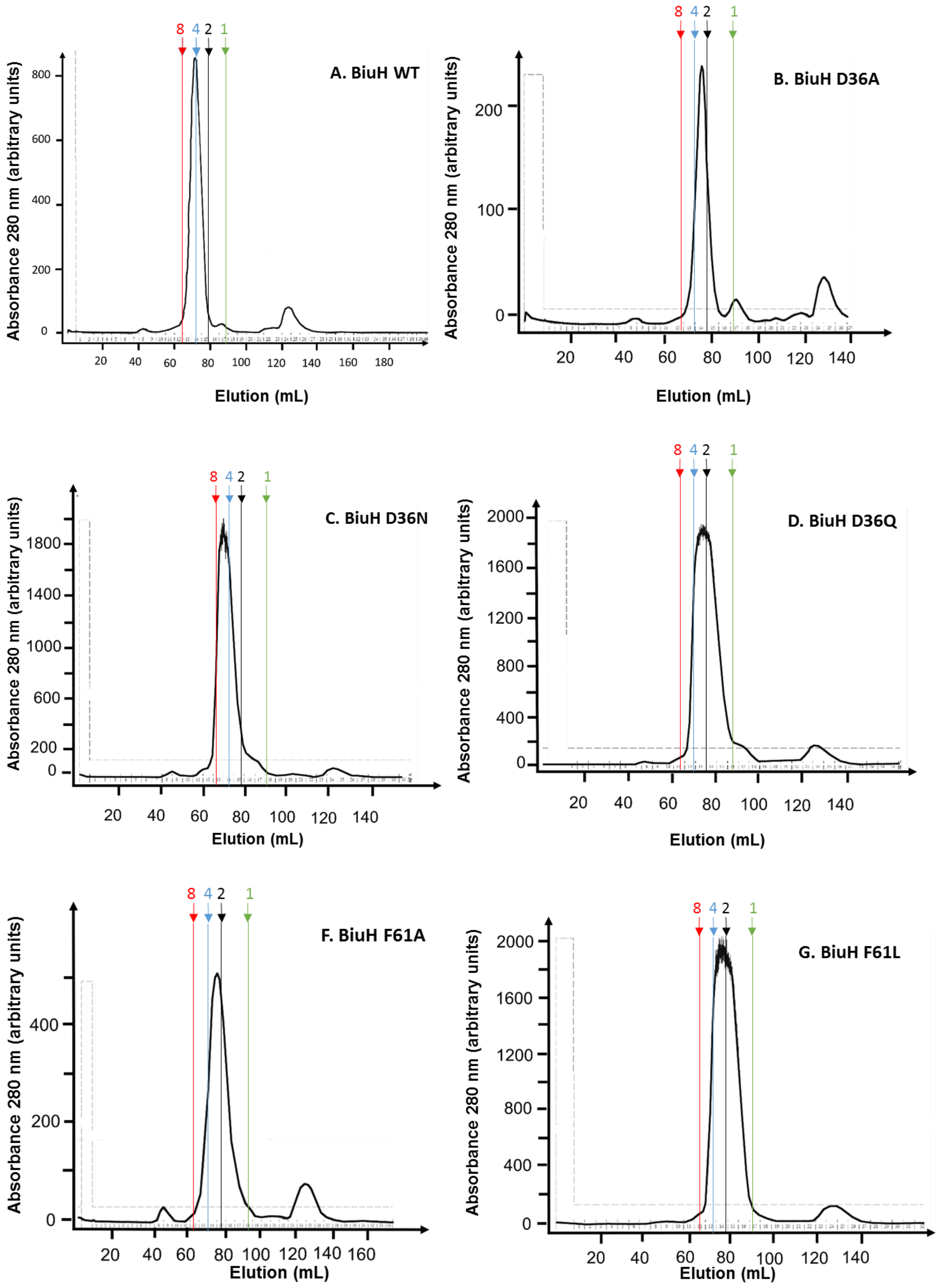

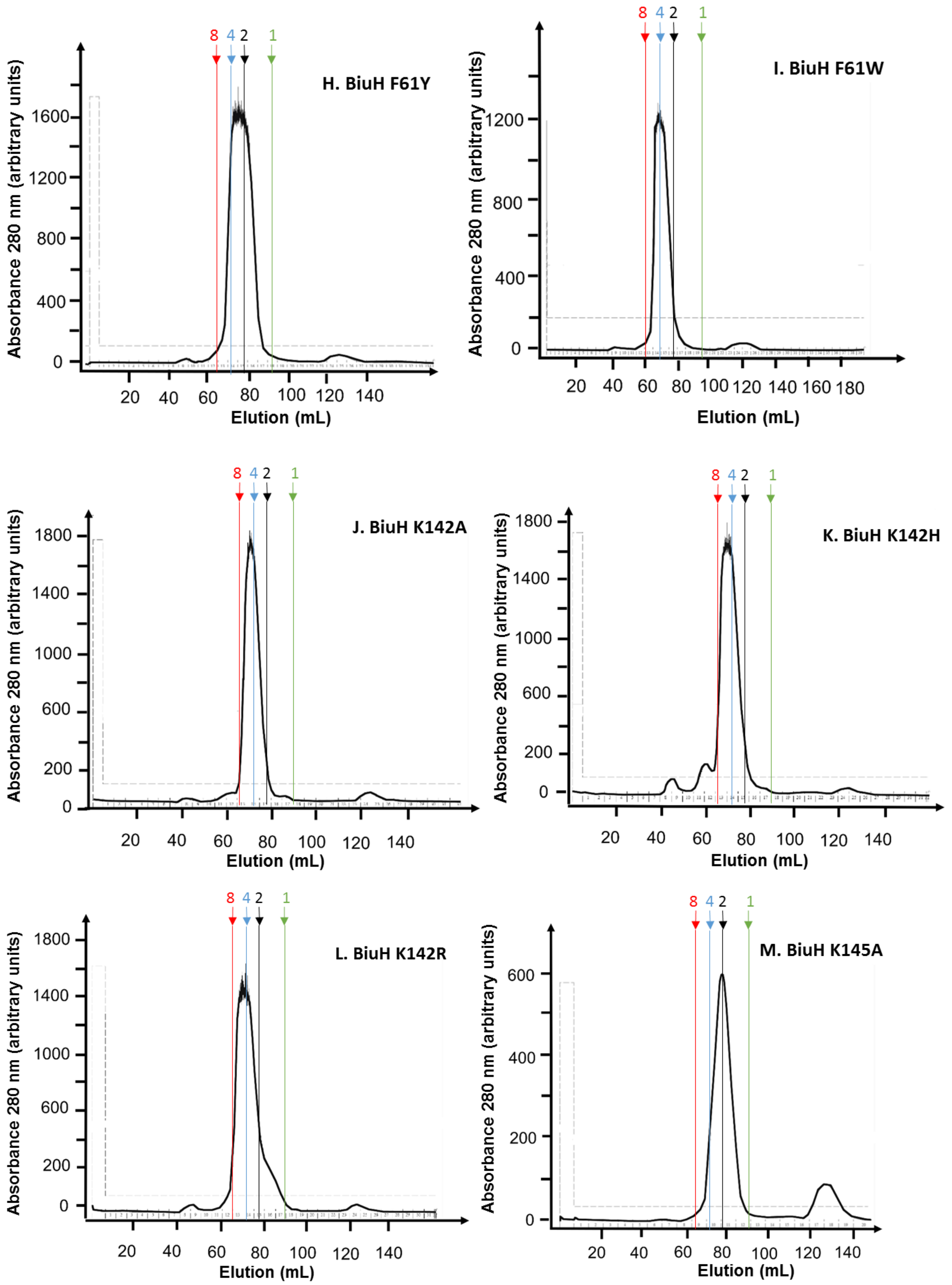
H. BiuH F61Y
I. BiuH F61W
J. BiuH K142A
K. BiuH K142H
L. BiuH K142R
M. BiuH K145A
8
4
2
1
Absorbance 280 nm (arbitrary units)
Elution (mL)
0
200
400
600
800
1000
1200
1400
1600
1800
20
40
60
80
100
120
140
160
180

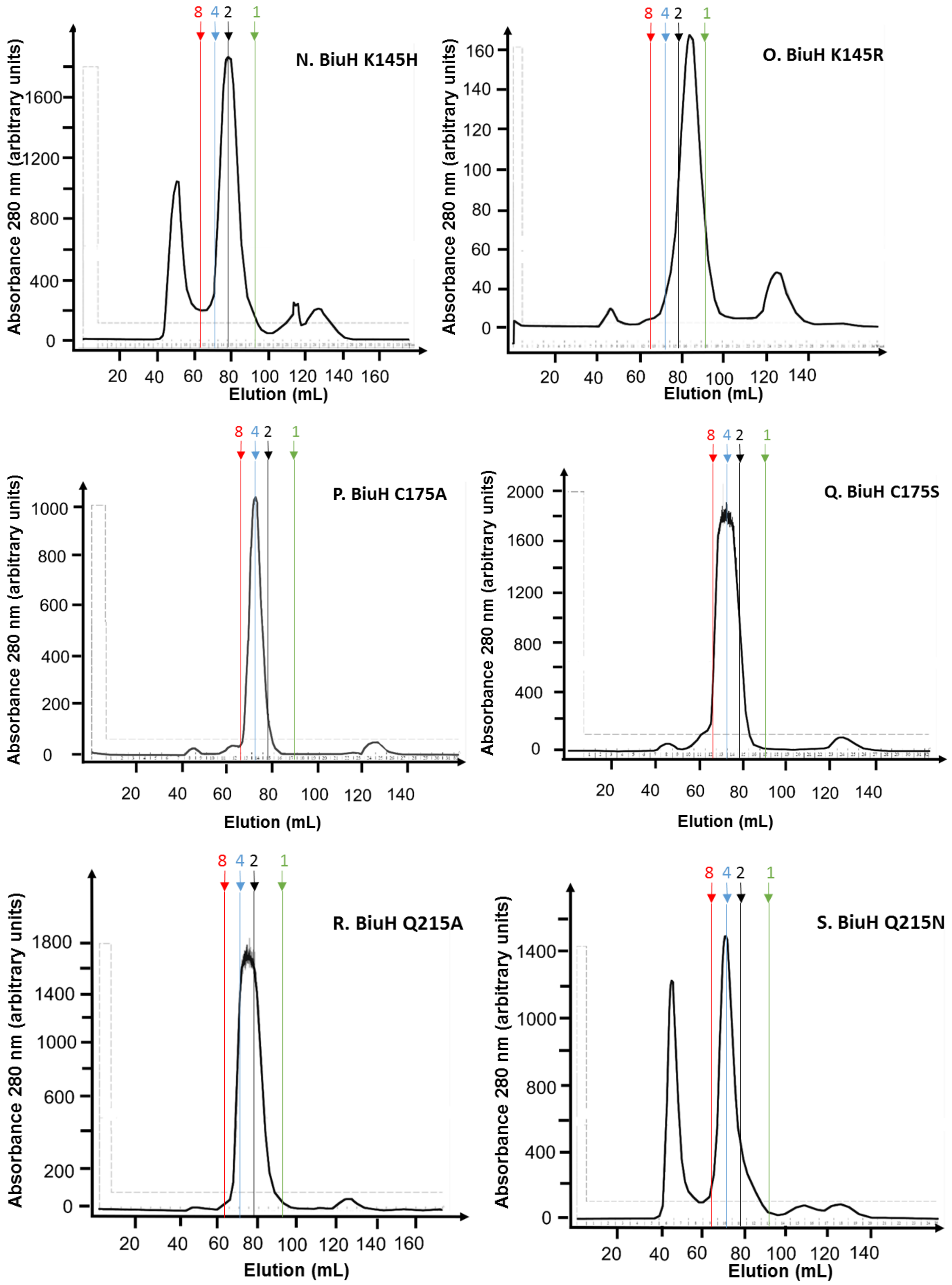

N. BiuH K145H
O. BiuH K145R
P. BiuH C175A
Q. BiuH C175S
R. BiuH Q215A
S. BiuH Q215N
8
4
2
1
Absorbance 280 nm (arbitrary units)
Elution (mL)

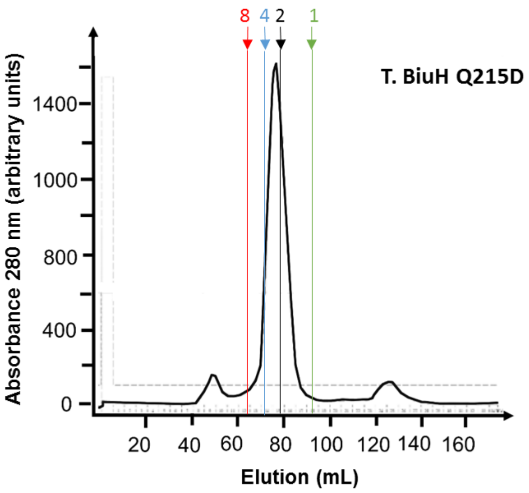
8
4
2
1
T. BiuH Q215D
Absorbance 280 nm (arbitrary units)
1400
1000
800
400
0
20
40
60
80
100
120
140
160
Elution (mL)

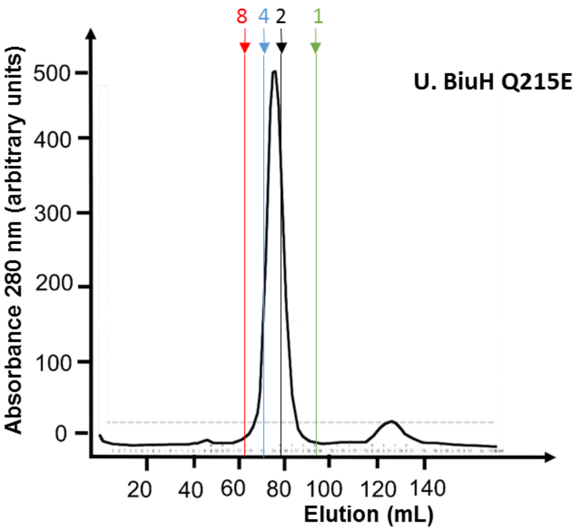
8
4
2
1
U. BiuH Q215E
Absorbance 280 nm (arbitrary units)
500
400
300
200
100
0
20
40
60
80
100
120
140
Elution (mL)
