# Supplementary material for: Structural and biochemical characterization of the biuret hydrolase (BiuH) from the cyanuric acid catabolism pathway of Rhizobium leguminasorum bv. viciae 3841
Source: PLoS One. 2018 Feb 9;13(2):e0192736. doi: 10.1371/journal.pone.0192736 (PMC5806882; doi:10.1371/journal.pone.0192736)

S8 Fig. Root mean square distance for BiuH active site and biuret during molecular dynamics simulation. Top: Root mean square distance from the crystallographic conformation for active site residues and biuret over the course of a 500 ns molecular dynamics simulation. Bottom: Distributions of root mean square distances from the crystallographic conformation for active side residues during a 500 ns molecular dynamics simulation.

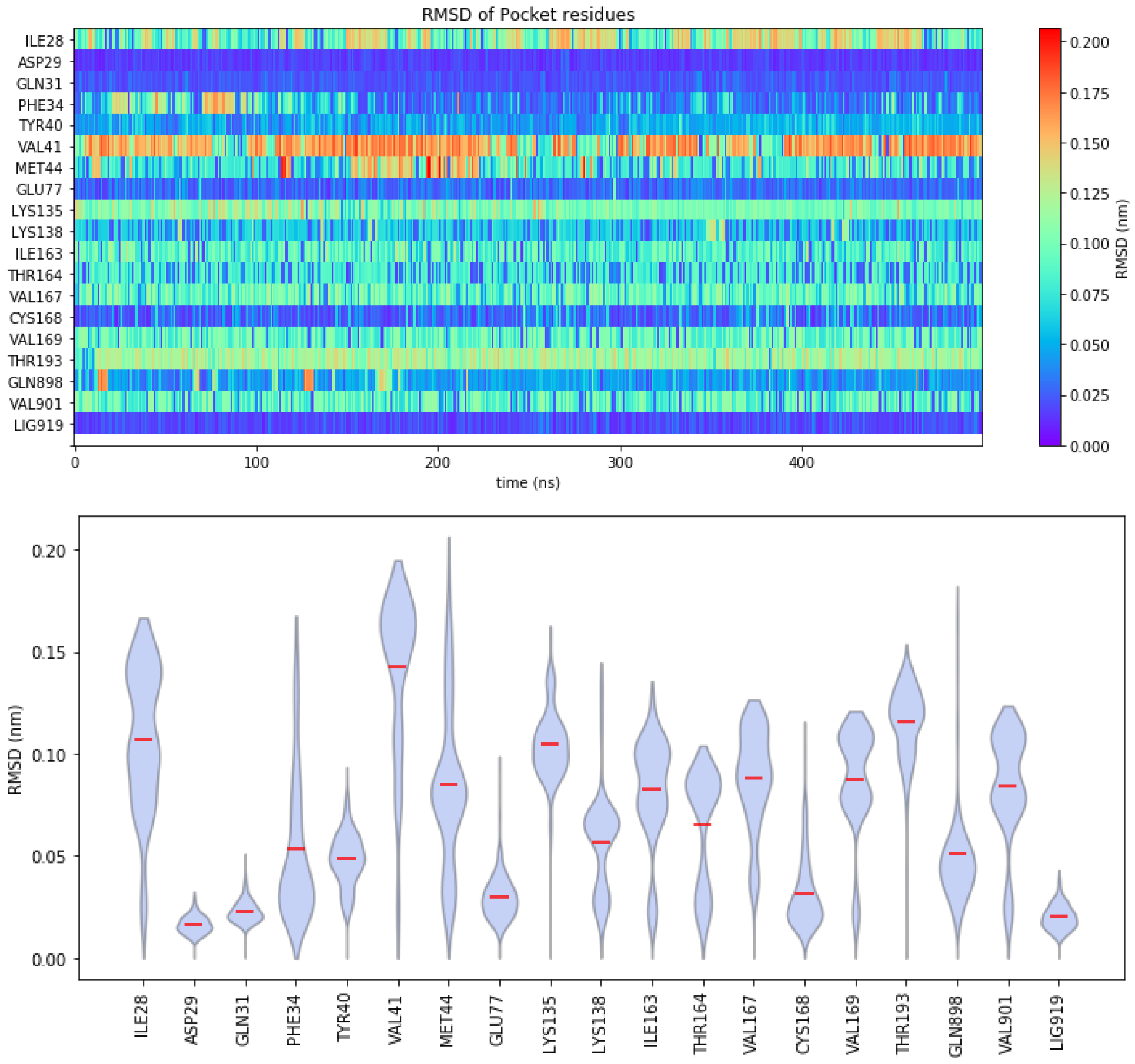

Supplement: S8 Fig — Top: Root mean square distance from the crystallographic conformation for active site residues and biuret over the course of a 500 ns molecular dynamics simulation. Bottom: Distributions of root mean square distances from the crystallographic conformation for active side residues during a 500 ns molecular dynamics simulation. (PDF) [file pone.0192736.s008.pdf]
