# Supplementary material for: Structural and biochemical characterization of the biuret hydrolase (BiuH) from the cyanuric acid catabolism pathway of Rhizobium leguminasorum bv. viciae 3841
Source: PLoS One. 2018 Feb 9;13(2):e0192736. doi: 10.1371/journal.pone.0192736 (PMC5806882; doi:10.1371/journal.pone.0192736)

**S9 Fig. Conformational clustering of active site residues throughout a 500ns molecular dynamics simulation.** States were assigned by clustering the Cartesian coordinates of the active site residues and biuret using the k-means algorithm (k = 20). Top: Discrete trajectory showing the conformational state of the active site throughout the course of the simulation. Bottom: Network diagram showing Markovian state model (lag time = 40 ns) of the conformational transitions between clustered states. The area of each node is proportional to the equilibrium probability of the state while the thickness of the arrows is proportional to the transition probability.

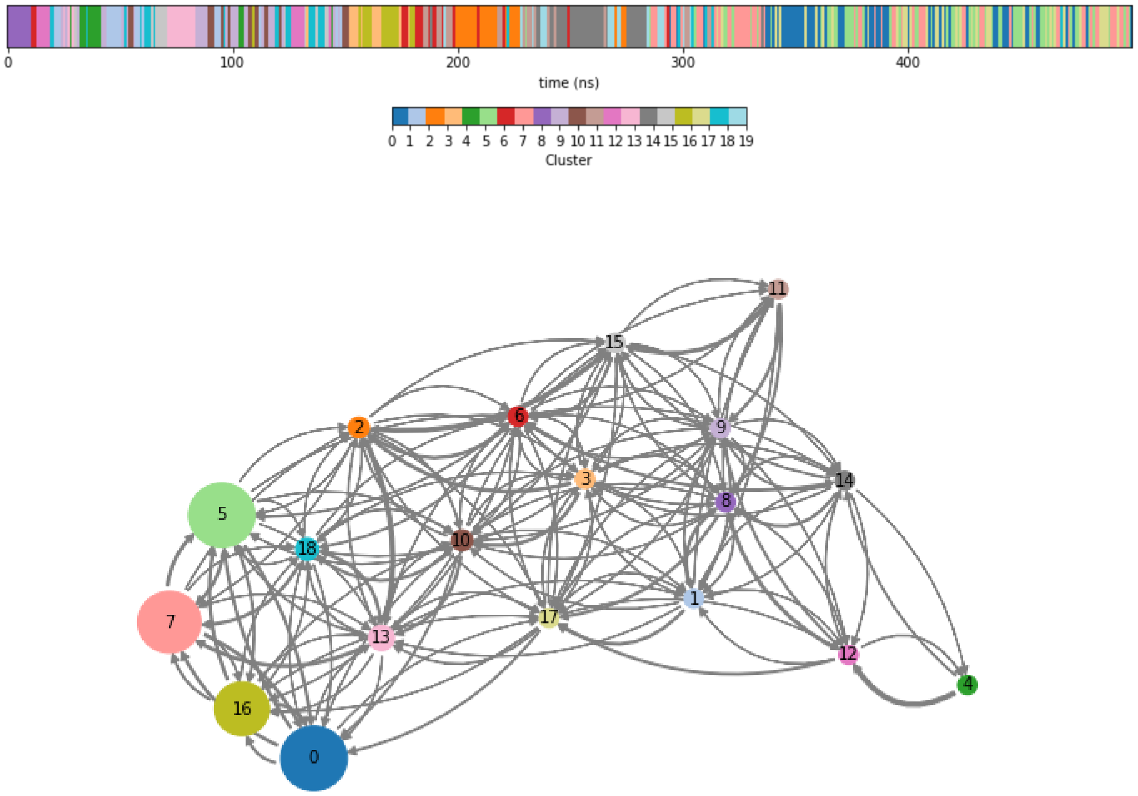

Supplement: S9 Fig — States were assigned by clustering the Cartesian coordinates of the active site residues and biuret using the k-means algorithm (k = 20). Top: Discrete trajectory showing the conformational state of the active site throughout the course of the simulation. Bottom: Network diagram showing Markovian state model (lag time = 40 ns) of the conformational transitions between clustered states. The area of each node is proportional to the equilibrium probability of the state while the thickness of the arrows is proportional to the transition probability. (PDF) [file pone.0192736.s009.pdf]
