# Supplementary material for: Structural and biochemical characterization of the biuret hydrolase (BiuH) from the cyanuric acid catabolism pathway of Rhizobium leguminasorum bv. viciae 3841
Source: PLoS One. 2018 Feb 9;13(2):e0192736. doi: 10.1371/journal.pone.0192736 (PMC5806882; doi:10.1371/journal.pone.0192736)

S10 Fig. Comparison of the biuret–enzyme interactions observed during molecular dynamics simulations for the WT enzyme (blue) and the C175S mutant (orange). The top row of plots shows the time trace of the number of hydrogen bonds between the active site residues and biuret, a histogram of these hydrogen bonds, and a bar chart highlighting differences in hydrogen bonding between WT and mutant. On the bottom, the distance between the nucleophile and the electrophilic carbon of biuret is shown as a function of time on the left, and distributions on the right.

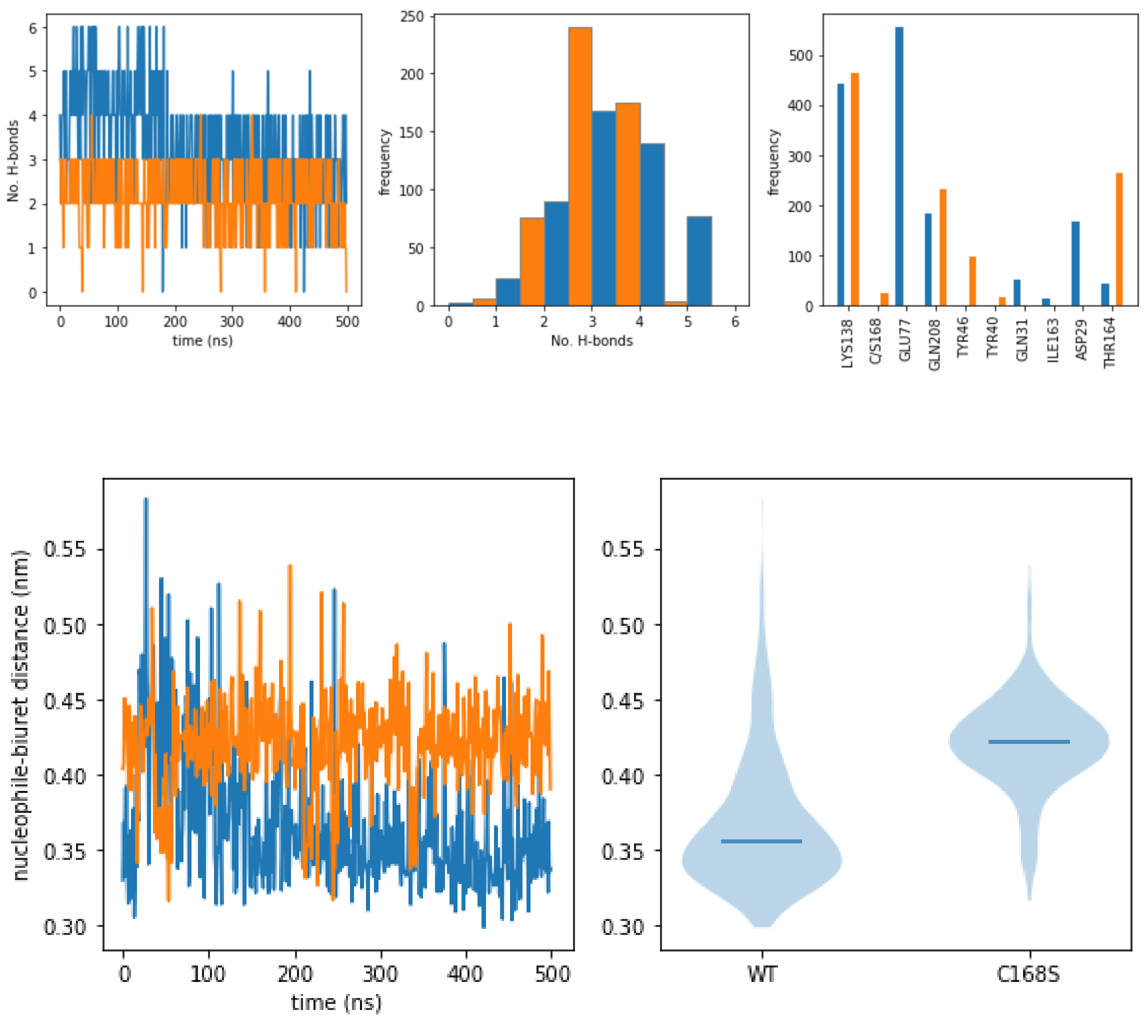

Supplement: S10 Fig — The top row of plots shows the time trace of the number of hydrogen bonds between the active site residues and biuret, a histogram of these hydrogen bonds, and a bar chart highlighting differences in hydrogen bonding between WT and mutant. On the bottom, the distance between the nucleophile and the electrophilic carbon of biuret is shown as a function of time on the left, and distributions on the right. (PDF) [file pone.0192736.s010.pdf]
