# Supplementary material for: Structural and biochemical characterization of the biuret hydrolase (BiuH) from the cyanuric acid catabolism pathway of Rhizobium leguminasorum bv. viciae 3841
Source: PLoS One. 2018 Feb 9;13(2):e0192736. doi: 10.1371/journal.pone.0192736 (PMC5806882; doi:10.1371/journal.pone.0192736)

Fig S11: Difference density map (Fo –Fc) shown around Biuret and the N-Carbamoyl-D,L-Aspartic acid inhibitor. A. Active site of the Cys175Ser BiuH variant shown in cartoon, with the active site amino acids and biuret shown in stick and the difference density map (Fo –Fc) in green mesh.
B. Active site of the Lys142Ala variant of BiuH showing Cys175 bound to the inhibitor N-carbamoyl-D,L-aspartic acid and the difference density map (F o –F c), with the active site amino acids and the inhibitor shown in stick and the difference density map (Fo –Fc) in green mesh.

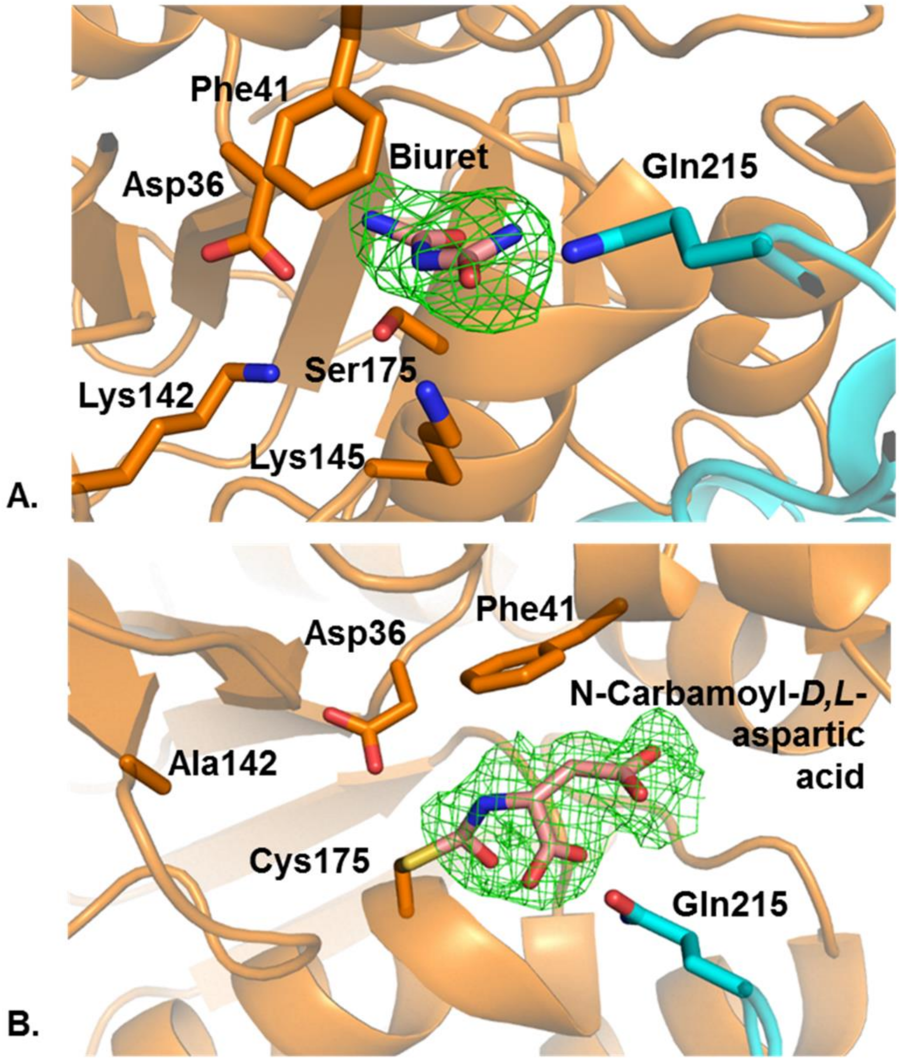

Supplement: S11 Fig — A. Active site of the Cys175Ser BiuH variant shown in cartoon, with the active site amino acids and biuret shown in stick and the difference density map (Fo −Fc) in green mesh. B. Active site of the Lys142Ala variant of BiuH showing Cys175 bound to the inhibitor N-carbamoyl-D,L-aspartic acid and the difference density map (Fo −Fc), with the active site amino acids and the inhibitor shown in stick and the difference density map (Fo −Fc) in green mesh. (PDF) [file pone.0192736.s011.pdf]
