# Supplementary material for: Structural and biochemical characterization of the biuret hydrolase (BiuH) from the cyanuric acid catabolism pathway of Rhizobium leguminasorum bv. viciae 3841
Source: PLoS One. 2018 Feb 9;13(2):e0192736. doi: 10.1371/journal.pone.0192736 (PMC5806882; doi:10.1371/journal.pone.0192736)
Supplement: S1 Table — In bold are the base pairs causing the mutation. (PDF) [file pone.0192736.s012.pdf]

S1 Table: Mutagenic primers used for introducing point mutation in BiuH's sequence by overlapping PCR. In bold are the base pairs causing the mutation.

Amino acid mutated	Mutagenic primer 5' → 3'
Asp36Ala Fwd	gccctcatcatcatc**gcc**atgcagacggatttc
Asp36Ala Rev	gaaatccgtctgcat**ggc**gatgatgatgagggc
Asp36Asn Fwd	ccgccctcatcatcatc**aac**atgcagacggatttc
Asp36Asn Rev	gaaatccgtctgcatg**ttg**atgatgatgagggcgg
Asp36Gln Fwd	ccgccctcatcatcatc**cag**atgcagacggatttctg
Asp36Gln Rev	cagaaatccgtctgcat**ctg**gatgatgatgagggcgg
Asp36Glu Fwd	gccctcatcatcatc**gag**atgcagacggatttctg
Asp36Glu Rev	cagaaatccgtctgcat**ctc**gatgatgatgagggc
Lys142Ala Fwd	gagacgatcatcgac**gcg**cccggcaagggttc
Lys142Ala Rev	gaacccttgccggg**cgc**gtcgatgatcgtctc
Lys142His Fwd	gagacgatcatcgac**cat**cccggcaagggttcg
Lys142His Rev	cgaacccttgccggg**atg**gtcgatgatcgtctc
Lys142Arg Fwd	ggcgagacgatcatcgac**agg**cccggcaag
Lys142Arg Rev	cttgccggg**cct**gtcgatgatcgtctcgcc
Cys175Ala Fwd	gatcaccaccgatgt**cgc**cgtctcgacgacgatg
Cys175Ala rev	catcgtcgtcgagacg**gcg**acatcggtggtgatc
Cys175Ser Fwd	caccaccgatgtc**agc**gtctcgacgac
Cys175Ser rev	gtcgtcgagac**gct**gacatcggtggtg
Lys145Ala Fwd	tcgacaagcccgg**cgc**gggttcgttctgcg
Lys145Ala Rev	cgcagaacgaaccc**gcg**ccgggcttgtcga
Lys145His Fwd	tcgacaagcccggc**cat**ggttcgttctgcgc
Lys145His Rev	gcgcagaacgaacc**atg**gccgggcttgtcga
Lys145Arg Fwd	atcgacaagcccggc**agg**ggttcgttctg
Lys145Arg Rev	cagaacgaacc**cct**gccgggcttgtcgat
Gln215Ala Fwd	atcaagatggtgaagatg**gcg**ggcggcgtcttcg
Gln215Ala Rev	cgaagacgccgcc**cgc**catcttcaccatcttgat
Gln215Asn Fwd	caagatggtgaagatg**aat**ggcggcgtcttcggc
Gln215Asn Rev	gccgaagacgccgcc**att**catcttcaccatcttg
Gln215Asp Fwd	caagatggtgaagatg**gat**ggcggcgtcttcggc
Gln215Asp Rev	gccgaagacgccgcc**atc**catcttcaccatcttg
Gln215Glu Fwd	tcaagatggtgaagatg**gag**ggcggcgtct
Gln215Glu Rev	agacgccgcc**ctc**catcttcaccatcttga
Phe41Ala Fwd	catcatcgacatgcagacggat**gcc**tgcggcaaggg
Phe41Ala Rev	cccttgccgca**ggc**atccgtctgcatgtcgatgatg
Phe41Leu Fwd	catcgacatgcagacggat**tta**tgcggcaaggg
Phe41Leu Rev	cccttgccgca**taa**atccgtctgcatgtcgatg
Phe41Tyr Fwd	catcatcgacatgcagacggat**tat**tgcggcaagggc
Phe41Tyr Rev	gcccttgccgca**ata**atccgtctgcatgtcgatgatg
Phe41Trp Fwd	tcatcgacatgcagacggat**tgg**tgcggcaaggg
Phe41Trp Rev	cccttgccgca**cca**atccgtctgcatgtcgatga
