# Supplementary material for: Structural and biochemical characterization of the biuret hydrolase (BiuH) from the cyanuric acid catabolism pathway of Rhizobium leguminasorum bv. viciae 3841
Source: PLoS One. 2018 Feb 9;13(2):e0192736. doi: 10.1371/journal.pone.0192736 (PMC5806882; doi:10.1371/journal.pone.0192736)
Supplement: S2 Table — The specific activity was measured in presence of 1.2 mM of biuret (n = 3) in μmoles.sec-1.mg enzyme-1, Tm: melting temperature measured by differential scanning fluorimetry in °C (n = 3–16 depending on the variants). (PDF) [file pone.0192736.s013.pdf]

S2 Table: Specific activity of BiuH and its variants. The specific activity was measured in presence of 1.2 mM of biuret (n=3) in µmoles.sec^{-1}.mg $enzyme^{-1}$, Tm: melting temperature measured by differential scanning fluorimetry in °C (n=3-16 depending on the variants).

	**Specific activity**	**Tm (°C)**
**BiuH WT**	51.02 ± 9.670	58.0 ± 0.45
**Asp36Ala**	0.05 ± 0.012	53.3 ± 0.16
**Asp36Asn**	0.03 ± 0.006	46.6 ± 0.05
**Asp36Gln**	0.05 ± 0.009	45.9 ± 0.09
**Asp36Glu**	0.04 ± 0.087	41.3 ± 0.07
**Phe41Ala**	1.79 ± 0.033	47.8 ± 0.31
**Phe41Leu**	6.28 ± 0.057	54.4 ± 0.41
**Phe41Tyr**	15.33 ± 0.068	59.7 ± 0.07
**Phe41Trp**	6.84 ± 0.082	51.9 ± 0.24
**Lys142Ala**	0.04 ± 0.015	64.3 ± 2.21
**Lys142His**	0.05 ± 0.009	67.8 ± 0.08
**Lys142Arg**	2.75 ± 0.466	45.8 ± 0.22
**Lys145Ala**	0.23 ± 0.004	53.1 ± 0.31
**Lys145His**	0.55 ± 0.074	54.5± 1.13
**Lys145Arg**	0.34 ± 0.04	47.5 ± 0.19
**Cys175Ala**	0.04 ± 0.032	61.0 ± 0.73
**Cys175Ser**	0.05 ± 0.011	64.5 ± 0.15
**Gln215Ala**	2.22 ± 0.22	51.7 ± 0.24
**Gln215Asn**	1.92 ± 0.08	51.9 ± 0.13
**Gln215Asp**	0.22 ± 0.007	52.2 ± 0.24
**Gln215Glu**	0.87 ± 0.081	56.0 ± 0.51
