# Supplementary material for: Structural and biochemical characterization of the biuret hydrolase (BiuH) from the cyanuric acid catabolism pathway of Rhizobium leguminasorum bv. viciae 3841
Source: PLoS One. 2018 Feb 9;13(2):e0192736. doi: 10.1371/journal.pone.0192736 (PMC5806882; doi:10.1371/journal.pone.0192736)
Supplement: S3 Table — (PDF) [file pone.0192736.s014.pdf]

S3 Table: Crystallisation conditions

PDB code	Protein variant	Protein details	Treatment	Additions	Crystallisation condition	Temperature
**6AZO**	SeMet native	4mg/mL in 50 mM tris pH 8<br>50 mM NaCl<br>5 mM DTT	chymotrypsin		0.1 M bis-tris chloride pH 6.14<br>0.089 M lithium sulfate<br>20.7 %w/v polyethylene glycol 4000	8 °C
**6AZN**	C175S	13 mg/mL in 50 mM tris pH 7.5<br>100 mM NaCl	thrombin	5 mM biuret (from 500 mM stock in DMSO)	0.1 M citrate buffer pH 5.5<br>0.02 M calcium chloride<br>12 %w/v polyethylene glycol 8000	20 °C
**6AZQ**	C175S	13 mg/mL in 50 mM tris pH 7.5<br>100 mM NaCl	thrombin	Saturated biuret in 50 mM tris pH 7.5, 100 mM NaCl	0.1 M citrate buffer pH 5.46<br>0.027 M calcium chloride<br>11.9 %w/v polyethylene glycol 8000	20 °C
**6AZS**	K142A	7 mg/mL in 50 mM tris pH 7.5<br>100 mM NaCl	thrombin	1 mM N-carbamoyl DL aspartic acid (from 100 mM stock in DMSO)	0.1 M bis tris chloride pH 6.09<br>23.6 %w/v polyethylene glycol 3350<br>0.161 M sodium thiocyanate	8 °C
**5BK6**	K142H	100 mg/mL in 50 mM tris pH 7.5<br>100 mM NaCl			0.1 M bis tris chloride pH 5.5<br>17 %w/v polyethylene glycol 10000<br>0.1 M sodium acetate	20 C
